# A Structurally Characterized Cobalt(I) σ‐Alkane Complex

**DOI:** 10.1002/anie.201914940

**Published:** 2020-02-20

**Authors:** Timothy M. Boyd, Bengt E. Tegner, Graham J. Tizzard, Antonio J. Martínez‐Martínez, Samuel E. Neale, Michael A. Hayward, Simon J. Coles, Stuart A. Macgregor, Andrew S. Weller

**Affiliations:** ^1^ Chemistry Research Laboratories Department of Chemistry University of Oxford Oxford OX1 3TA UK; ^2^ Department of Chemistry University of York York YO10 5DD UK; ^3^ Institute of Chemical Sciences Heriot-Watt University Edinburgh EH14 4AS UK; ^4^ UK National Crystallography Service Chemistry Faculty of Engineering and Physical Sciences University of Southampton Southampton SO17 1BJ UK

**Keywords:** alkane complexes, cobalt, periodic density functional theory, single crystals, X-ray diffraction

## Abstract

A cobalt σ‐alkane complex, [Co(Cy_2_P(CH_2_)_4_PCy_2_)(norbornane)][BAr^F^_4_], was synthesized by a single‐crystal to single‐crystal solid/gas hydrogenation from a norbornadiene precursor, and its structure was determined by X‐ray crystallography. Magnetic data show this complex to be a triplet. Periodic DFT and electronic structure analyses revealed weak C−H→Co σ‐interactions, augmented by dispersive stabilization between the alkane ligand and the anion microenvironment. The calculations are most consistent with a η^1^:η^1^‐alkane binding mode.

## Graphical abstract

**A cobalt σ‐alkane complex** was synthesized by a single‐crystal to single‐crystal solid/gas hydrogenation from a norbornadiene precursor, and its structure was determined by X‐ray crystallography. Periodic DFT and electronic structure analyses revealed weak C−H→Co σ‐interactions, augmented by dispersive stabilization between the alkane ligand and the anion microenvironment.

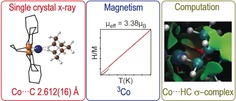

In σ‐alkane complexes, a C−H group interacts with a metal in a three‐center two‐electron bond (3c‐2e), and these species are key intermediates in stoichiometric and catalytic hydrocarbon C−H activation processes.^1^, ^2^ The directional, non‐nucleophilic, and strong C−H bond means that alkanes are poor ligands (40–60 kJ mol^−1^), making σ‐alkane complexes challenging to observe because of displacement of the alkane by solvent or other ligands.^3^ Elegant methods have thus been developed to generate and characterize σ‐alkane complexes in the solution phase (Figure 1 A). Low‐temperature NMR spectroscopy, using in situ photolysis or protonolysis, has revealed σ‐alkane complexes such as **1** 4 and **2**,^5^ respectively, while fast time‐resolved spectroscopic studies (TRIR)^6^ have probed the formation and onward reactivity of transient σ‐alkane complexes (e.g., **3**).^7^, ^8^

**Figure 1:**
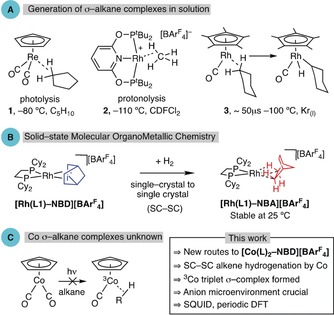
A) Examples of σ‐alkane complexes, generated using in situ photolysis (**1**) or protonation (**2**), and as intermediates in C−H activation (**3**). B) Single‐crystal to single‐crystal generation of a Rh σ‐alkane complex by hydrogenation of a diene. C) Instability of Co σ‐alkane complexes.

We have recently developed molecular solid‐state routes to σ‐alkane complexes that circumvent the need for solvent. By using single‐crystal to single‐crystal (SC‐SC) gas/solid reactivity,^9^, ^10^ synthetically significant amounts (up to ca. 1 g) of σ‐alkane complexes such as [Rh(**L1**)‐NBA][BAr^F^_4_] (Figure 1 B; NBA=norbornane, Ar^F^=3,5‐(CF_3_)_2_C_6_H_3_, **L1**=Cy_2_PCH_2_CH_2_PCy_2_) can be formed by simple hydrogenation of a cationic diene precursor (norbornadiene, NBD). These complexes can show remarkable stabilities (months, 25 °C),^11^ allowing for their full characterization (single‐crystal X‐ray diffraction, solid‐state NMR spectroscopy) and studies into alkane mobility and reactivity.^10^, ^12^, ^13^ Their isolation comes, in large part, from the stabilizing [BAr^F^_4_]^−^ anion microenvironment, which often forms an approximately octahedral cage surrounding the metal cation. We have termed this methodology solid‐state molecular organometallic (SMOM) chemistry.^12^

Despite these advances in both solution and solid‐state chemistries, there remain significant challenges. One of these is that while the heavier congeners of Group 9 (Rh and Ir) have offered a rich hunting ground for the generation of σ‐alkane complexes and subsequent catalytic C−H activation,^2^, ^3^, ^7^, ^14^ no cobalt examples are known. Indeed, the only σ‐alkane complex of cobalt thus far reported in the literature is Co(CH_4_), generated using isolated Co atoms in an Ar matrix at −261 °C.^15^ While the 3d metal cobalt is expected to form weak 3c‐2e bonds with alkanes, an additional problem is the accessibility of the triplet ^3^Co spin state, which further discourages the formation of strong bonds. For example, while the photogenerated {^1^Rh(CO)Cp*} singlet fragment forms a transient σ‐alkane complex (**3**),^7^ no such complexes are observed for equivalent {Co(CO)Cp} (Figure 1 C).^16^ Calculations show that a ^3^Co(CO)Cp⋅⋅⋅HCH_3_ interaction would be repulsive, and while ^1^Co(CO)Cp⋅⋅⋅HCH_3_ is accessible, its formation is endergonic with respect to the reactants.^17^ In contrast, ^3^Mn(CO)_2_Cp(heptane) has been implicated as a transient intermediate on the pathway to C−H activation based on TRIR, DFT calculations, and kinetic modeling.^18^ σ‐H_2_ complexes of cobalt are known.^19^

We now report that, by harnessing the stabilizing microenvironment of the [BAr^F^_4_]^−^ anions with a cationic [Co(diphosphine)‐NBD]^+^ precursor, we have been able to structurally characterize a very weakly bound Co σ‐alkane complex using SMOM techniques. This complex also has a ^3^Co triplet spin state, as probed by magnetic measurements and computation.

[Rh(**L**)‐NBD][BAr^F^_4_] is a versatile motif for the generation of σ‐alkane complexes by SC‐SC transformations (**L**=Cy_2_P(CH_2_)_*n*_PCy_2_).^9^, ^11^ We have thus developed a route to the equivalent cobalt(I) complexes of these Schrock–Osborn^20^ systems (Figure 2; *n*=2, **L1**; *n*=4, **L2**). This starts from CoCl_2_, involves a KC_8_ reduction, a CH_2_Cl_2_ extraction, and delivers, respectively, lilac and green crystalline products in moderate yield.^21^ Our method complements that recently reported by Chirik using DuPhos‐based ligands.^22^ [Co(**L1**)(arene)][BF_4_] complexes are also known.^23^ The solid‐state structure of [Co(**L2**)‐NBD][BAr^F^_4_] is shown in Figure 2, with that for [Co(**L1**)‐NBD][BAr^F^_4_] given in the Supporting Information. Both show a cobalt coordination sphere that is twisted from square‐planar towards tetrahedral. This distortion is greater with a wider bite angle ligand in [Co(**L2**)‐NBD][BAr^F^_4_] (28.0(3)° vs. 22.5(2)°).^24^ The [BAr^F^_4_]^−^ anions adopt an approximately *O*_h_ motif in the solid state, being very similar to the direct analogues [Rh(**L**)‐NBD][BAr^F^_4_].^9^, ^11^

**Figure 2:**
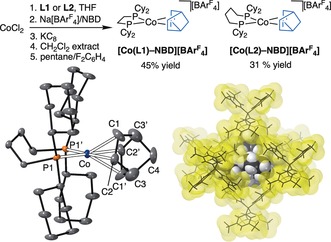
Synthesis of cationic Co‐NBD precursors. **L1**=Cy_2_P(CH_2_)_2_PCy_2_, **L2**=Cy_2_P(CH_2_)_4_PCy_2_. Solid‐state structure of the cationic portion of [Co(**L2**)‐NBD][BAr^F^_4_]; 15 % displacement ellipsoids, anion and H atoms not shown, −173 °C data collection.^34^ Approximately octahedral packing of [BAr^F^_4_]^−^ anions around a single cation. Surfaces shown at van der Waals radii. Selected bond lengths [Å] and angles [°]: Co–C1 2.212(6), Co–C2 2.110(4), C1–C2 1.394(6), Co–P1 2.2717(8); P‐Co‐P1 103.09(4); ∡CoP1P1′/Co(centC1C2)(centC1′C2′) 28.0(3).

In FC_6_H_5_ solution, [Co(**L1**)‐NBD][BAr^F^_4_] reacts quantitatively with H_2_ to form (in 90 % isolated yield) the brown, crystallographically characterized^21^ arene adduct [Co(**L1**)(η‐FC_6_H_5_)][BAr^F^_4_], with the concomitant formation of norbornane. This result shows that these cationic Co^I^ systems will promote the hydrogenation of alkenes,^25^ similar to the DuPhos variants.^22^ For [Co(**L2**)‐NBD][BAr^F^_4_], hydrogenation in FC_6_H_5_ solution results in decomposition to a mixture of products. However, as we,^12^, ^26^ and others,^27^ have previously reported, organometallic synthesis in the solid state can promote desirable changes in selectivity when compared to solution routes. This is the case for [Co(**L2**)‐NBD][BAr^F^_4_].

Addition of H_2_ to single crystals of [Co(**L2**)‐NBD][BAr^F^_4_] results in the formation of the highly reactive σ‐alkane complex [Co(**L2**)‐NBA][BAr^F^_4_] in a SC‐SC transformation. By optimizing H_2_ addition time (2 bar, 1 h, 298 K), data collection parameters (2 h, −173 °C, frozen oil drop), and rapid transfer to the diffractometer, an acceptable refinement (*R1=*8 %) and unambiguous structural solution was achieved. [Co(**L2**)‐NBA][BAr^F^_4_] is so reactive that even at −173 °C data acquisition times longer than 2 h resulted in steady decomposition, loss of diffraction, and a color change of the crystal from brown to blue (i.e., Co^II^), which we suggest is due to reaction with adventitious oxygen in the mounting oil.

The solid‐state structure of [Co(**L2**)‐NBA]^+^ is presented in Figure 3 A. This shows a {Co(Cy_2_P(CH_2_)_4_PCy_2_}^+^ fragment, with crystallographically imposed *C*_2_ symmetry, in which the NBA ligand sits in a cleft formed between two [BAr^F^_4_]^−^ aryl rings in the approximately octahedral anion microenvironment, as noted for Rh analogues.^9^, ^11^ The phosphine backbone is disordered over two conformations, and only one is shown. Hydrogen atoms were placed in calculated positions. The metal center is weakly bound by a saturated NBA fragment through two, mutually opposing, *endo*‐Co⋅⋅⋅H−C interactions: Co⋅⋅⋅H(1A), 1.766 Å; Co⋅⋅⋅C1, 2.612(16) Å. The other carbon atoms in the NBA ligand are significantly further away [Co⋅⋅⋅C2, 2.92(2) Å]. The Co−P distances become slightly shorter on hydrogenation [2.232(1) Å vs. 2.2747(9) Å], suggesting a weaker *trans* ligand. As for the precursor, the NBA fragment is twisted away from square‐planar (∡CoP1P1′/CoC1C1′≈40°; see Figures S12 and S13).^21^ Overall this is different from the orientation found in [Rh(**L2**)‐NBA][BAr^F^_4_] (Figure 3 C), in which the alkane interacts through two adjacent η^2^‐*endo*‐Rh⋅⋅⋅H−C bonds at a pseudo‐square‐planar Rh^I^ center. The M⋅⋅⋅C distances are also approximately 0.22 Å longer than in [Rh(**L2**)‐NBA][BAr^F^_4_], which is a significant observation given the small difference in covalent radii between Rh^I^ and high‐spin Co^I^ (1.42 and 1.50 Å, respectively).^28^ While the atomic displacement parameters associated with the cobalt center, phosphine ligand, and anion are unremarkable, those of the NBA fragment are both significantly larger and show pronounced ellipticity. This signals that the alkane is, relatively, less constrained in the approximately octahedral [BAr^F^_4_]^−^ microenvironment. As a consequence of this, the C1−C2 distance in the NBA fragment was restrained to be in the range of a C−C bond, 1.470(12) Å. All of these data point to a very weakly bound σ‐alkane ligand at a Co^I^ center that is electronically very different from its Rh analogue. That this is a weak intermolecular interaction is signaled by the Co⋅⋅⋅C distances being much longer than found for the small number of structurally characterized Co⋅⋅⋅H−C intramolecular agostic complexes (ca. 2.2 Å),^29^ being more comparable to weak agostic M⋅⋅⋅H−C interactions (M=Rh, Ir, ca. 2.8 Å).^30^ While rare, σ‐alkane complexes of 3d metals have been characterized at low temperature by in situ NMR spectroscopy.^31^

**Figure 3:**
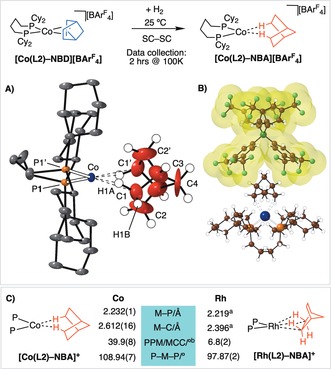
Synthesis of [Co(**L2**)‐NBA][BAr^F^_4_]. A) Solid‐state structure of the cation; 15 % displacement ellipsoids, anion and most H atoms not shown.^34^ Hydrogen atoms were placed in calculated positions. −173 °C data collection. Selected bond lengths [Å] and angles [°]: Co–P 2.232(1), Co–C1 2.612(16), C1–C2 1.470(12); P1‐Co‐P1′ 108.94(7); ∡CoP1P1′/CoC1C1′ 39.9(8). B) Relationship between the cation and the proximate anion, van der Waals surface shown. C) Comparison of selected structural metrics between [Co(**L2**)‐NBA][BAr^F^_4_] and [Rh(**L4**)‐NBA][BAr^F^_4_]. [a] Average distances. [b] Angle between planes.

The SC‐SC hydrogenation upon addition of H_2_ to [Co(**L2**)‐NBD][BAr^F^_4_] was confirmed by adding CD_2_Cl_2_ to [Co(**L2**)‐NBA][BAr^F^_4_] and vacuum transfer of the volatiles. This shows that NBA has been formed (^1^H NMR analysis), with no residual NBD observed. Addition of H_2_ to [Co(**L1**)‐NBD][BAr^F^_4_] resulted in complete loss of crystallinity, although NBA is—again—formed.

The magnetization data for both [Co(**L2**)‐NBD][BAr^F^_4_] and [Co(**L2**)‐NBA][BAr^F^_4_] can be fit by the Curie–Weiss law [*χ*=*C*/(*T*−*θ*)+*K*] over the temperature range 50≤*T*/*K*≤300 to yield values consistent with a ^3^Co spin state (Figures 4 and S14). Low‐temperature deviations from Curie–Weiss behavior below 50 K are attributed to intermolecular magnetic couplings. The observation of a temperature‐independent component to the susceptibility of both compounds is indicative of a small unquenched orbital contribution to the magnetization, and is consistent with the slightly elevated moments for both compounds, compared to expected spin‐only values: [Co(**L2**)‐NBD][BAr^F^_4_] *μ*_eff_=3.14 μ_B_; [Co(**L2**)‐NBA][BAr^F^_4_] *μ*_eff_=3.38 μ_B_; *S*=1, spin‐only *μ*_eff_=2.82 μ_B_). The slightly larger moment for the alkane complex is in line with NBA being a weaker‐field ligand than NBD. Consistent with the ^3^Co spin state for both complexes ^31^P{^1^H} SSNMR spectra are featureless, as are the EPR spectra.

**Figure 4:**
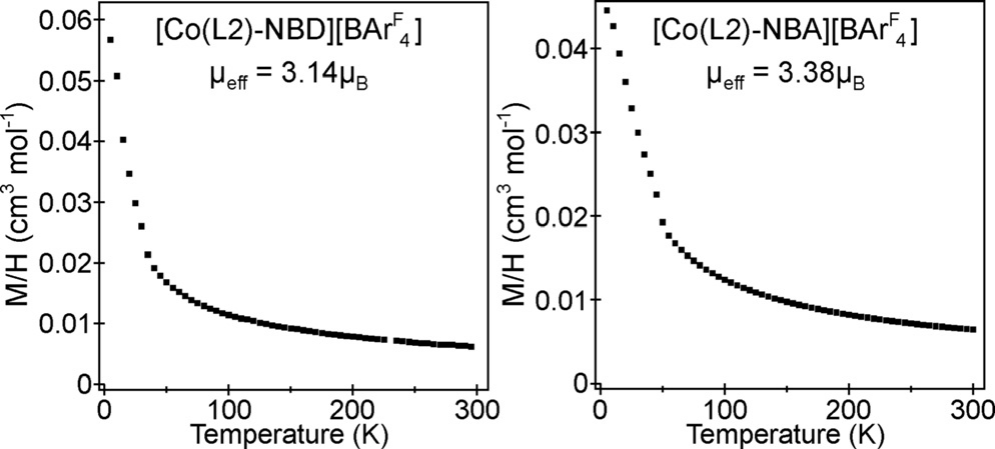
Magnetization data for [Co(**L2**)‐NBD][BAr^F^_4_] and [Co(**L2**)‐NBA][BAr^F^_4_]. See the Supporting Information for details.

Spin‐state energetics for [Co(**L2**)‐NBA][BAr^F^_4_] and [Co(**L2**)‐NBD][BAr^F^_4_] were also probed with periodic DFT calculations, with geometries based on the crystal structures and H and F positions optimized in the triplet state with the PBE‐D3 functional and energies recomputed with the hybrid PBE0‐D3 approach.^32^ For [Co(**L2**)‐NBA][BAr^F^_4_], the ^3^Co spin state is favored by 32.3 kcal mol^−1^ (at each Co center), consistent with the experimental magnetization data. In contrast, for [Co(**L2**)‐NBD][BAr^F^_4_], the ^3^Co spin state is only lower by 4.1 kcal mol^−1^, and further tests showed that the computed preferred spin state is highly sensitive to both methodology and NBD ligand orientation.^21^

The computed structure of the [Co(**L2**)‐NBA]^+^ cation (Figure 5 A, which also shows the labeling scheme adopted in the computational study 3) reveals a short Co⋅⋅⋅H11 distance of 1.82 Å, with an elongation of the C1−H11 bond to 1.12 Å, that together suggest a degree of C1−H11→Co σ‐interaction. In contrast, the Co⋅⋅⋅H21 distance is 2.58 Å and no elongation of the C2−H21 bond is seen. The QTAIM molecular graph (Figure 5 B) is consistent with these features and confirms a Co⋅⋅⋅H11 bond path. Figures 5 C and D show the NCI plot of the [Co(**L2**)‐NBA][BAr^F^_4_] ion pair, with weak interactions color‐coded from blue (most stabilizing) through green (weakly stabilizing) to red (destabilizing). Viewed from the Co center (Figure 5 C), the localized blue regions between Co⋅⋅⋅H11 and Co⋅⋅⋅H11′ suggest two η^1^‐C−H→Co σ‐interactions, with turquoise regions between Co⋅⋅⋅H21/H21′ reflecting weaker, dispersive stabilizations. The side‐on view of the ion pair (Figure 5 D) highlights broad regions of dispersive stabilization between the NBA ligand and i) the PCy_2_ substituents, ii) the aryl groups of the [BAr^F^_4_]^−^ anion, and iii) non‐classical C−F⋅⋅⋅H−C H‐bonding. These features, combined with the C−H→Co σ‐interactions, indicate how both inter‐ and intramolecular interactions contribute to stabilizing the alkane complex within the binding pocket.

**Figure 5:**
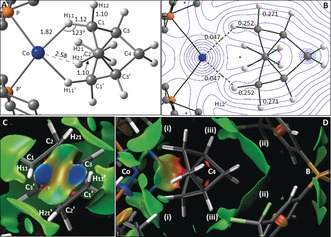
A) Computed structure of the [Co(**L2**)‐NBA]^+^ cation viewed down the C2⋅⋅⋅C2′ axis, highlighting key distances [Å] and angles to H atoms. B) Quantum theory of atoms in molecules (QTAIM) molecular graph with bond critical bonds (BCPs) in green, ring critical points (RCPs) in pink, and selected BCP electron densities, *ρ*(*r*), in e bohr^−3^. C) Non‐covalent interaction (NCI) plot of the proximate [Co(**L2**)‐NBA][BAr^F^_4_] ion pair viewed from the Co center. D) NCI plot perpendicular to the Co⋅⋅⋅C4⋅⋅⋅B axis, highlighting i) the PCy_2_ substituents, ii) the [BAr^F^_4_]^−^ aryl groups, and iii) C−F⋅⋅⋅H−C H‐bonding.

A spin‐unrestricted NBO second‐order perturbation analysis delineates interactions in the α‐ and β‐spins at the ^3^Co center (see Figure 6, which also gives the Lewis structure used). For α‐spin, occupation of all five 3d orbitals means donation from σ_C−H_ can only occur into a Co low‐valent (4s) NBO (3.5 kcal mol^−1^). A similar interaction is seen in the β‐spin (4.7 kcal mol^−1^), but donation can now also occur into a vacant Co d orbital (5.9 kcal mol^−1^). Back‐donation from P lone pairs into the σ*_C−H_ NBOs is seen for both spins, with additional back‐donation from an occupied Co 3d NBO in the α‐spin (1.7 kcal mol^−1^).^21^

**Figure 6:**
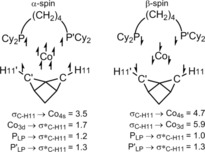
Spin‐unrestricted NBO second‐order perturbation analysis highlighting key donor–acceptor interactions [kcal mol^−1^] in [Co(**L2**)‐NBA]^+^. The resultant NLMOs are provided in the Supporting Information.

These NBO data indicate that NBA is less strongly bound in [Co(**L2**)‐NBA]^+^ than in the [Rh(**L2**)‐NBA]^+^ cation (where the total σ_C−H_ donation amounts to ca. 19.7 kcal mol^−1^ and back‐donation to σ*_C−H_ is ca. 8.5 kcal mol^−1[10, 33]^). This also aligns with the reduced C−H11 bond elongation and BCP *ρ*(*r*) of the ^3^Co system. The Co‐H11‐C angle of 123° along with the localized, stabilizing features along the C−H11/C′−H11′⋅⋅⋅Co vectors in the NCI plot suggest an η^1^:η^1^‐NBA binding mode in [Co(**L2**)‐NBA]^+^, again in contrast with the η^2^:η^2^‐NBA ligand in [Rh(**L2**)‐NBA]^+^.^10^ The NCI plot also reveals intermolecular dispersive interactions in this ^3^Co system, and these are likely to contribute relatively more to overall stability than in the related, more strongly covalently bound, ^1^Rh system.

The synthesis of [Co(**L2**)‐NBA][BAr^F^_4_] thus rests upon the stabilizing microenvironment provided by the [BAr^F^_4_]^−^ anions, underscoring the importance of such weak interactions in isolating σ‐alkane complexes in the solid state.^3^, ^9^ That this now also allows for the isolation of complexes that sit on a triplet surface opens up new opportunities for exploring the synthesis, reactivity, and catalysis of such species that have, at best, only a fleeting existence when generated in solution.^1^, ^18^

## Conflict of interest

The authors declare no conflict of interest.

## Supporting information

As a service to our authors and readers, this journal provides supporting information supplied by the authors. Such materials are peer reviewed and may be re‐organized for online delivery, but are not copy‐edited or typeset. Technical support issues arising from supporting information (other than missing files) should be addressed to the authors.

Supplementary
