# Supplementary material for: A Structurally Characterized Cobalt(I) σ‐Alkane Complex
Source: Angew Chem Int Ed Engl. 2020 Feb 20;59(15):6177–81. doi: 10.1002/anie.201914940 (PMC7187152; doi:10.1002/anie.201914940)
Supplement: Supplementary file 1 — Supplementary [file ANIE-59-6177-s001.pdf]

Angewandte Chemie
Eine Zeitschrift der Gesellschaft Deutscher Chemiker

Supporting Information

A Structurally Characterized Cobalt(I) σ-Alkane Complex

Timothy M. Boyd, Bengt E. Tegner, Graham J. Tizzard, Antonio J. Martínez-Martínez, Samuel E. Neale, Michael A. Hayward, Simon J. Coles, Stuart A. Macgregor, and Andrew S. Weller**

anie_201914940_sm_miscellaneous_information.pdf

Author Contributions

T.B. Investigation: Lead; Methodology: Equal.

Table of Contents

S.1. Experimental Details 2
  S.1.1. General Methods and Chemicals 2
  S.1.2. NMR Spectroscopic Data 3
  S.1.3. Mass Spectrometry Data 3
S.2. Synthetic Procedures and Characterisation data 4
  S.2.1. Preparation and Characterisation of [Co(**L1**)-NBD][BAr$^F_4$] 4
  S.2.2. Preparation and Characterisation of [Co(**L1**)(η-FC_6H_5)][BAr$^F_4$] 6
  S.2.3. Preparation and Characterisation of [Co(**L2**)-NBD][BAr$^F_4$] 9
  S.2.4. Preparation and Characterisation of [Co(**L2**)-NBA][BAr$^F_4$] 10
S.3. Crystallographic and Refinement Data 12
  S.3.1. Crystal Structure Determinations 12
  S.3.2. Additional Comments Crystal Structures and Refinement Data 12
  S.3.3. Single Crystal X-ray Diffraction Images 15
S.4. Magnetic Characterisation 19
S.5. Computational Methods 21
  S.5.1. Solid State Calculations 21
  S.5.2. Molecular Calculations 23
  S.5.3. Functional Testing and Spin Energetics. 24
S.6. Electronic Structure Analyses. 26
  S.6.1. [Co(**L2**)–NBA][BAr$^F_4$] 26
S.8. References 34

S.1. Experimental Details

S.1.1. General Methods and Chemicals

All manipulations (unless otherwise stated) were performed under an atmosphere of argon, using standard Schlenk techniques on a dual vacuum/inlet grease-free J. Young tap manifold. All new prepared and isolated organometallic compounds were stored in a MBraun glovebox under an atmosphere of argon. Glassware was dried in an oven at 140°C overnight prior to use. n-Pentane, n-hexane and dichloromethane (CH_2Cl_2, DCM) were all dried using an MBraun SPS-800 solvent purification system and degassed by three freeze-pump-thaw cycles. Anhydrous tetrahydrofuran (THF) was distilled under reflux with sodium metal and benzophenone under a nitrogen atmosphere and stored over activated 3 Å molecular sieves for 24 hours prior to use. Deuterated dichloromethane (CD_2Cl_2) was purchased from Cambridge Isotope Laboratories Inc, dried by stirring over CaH_2 overnight before being vacuum distilled, subsequently degassed by three freeze-pump-thaw cycles and then stored over activated 3 Å molecular sieves. 1,2-difluorobenzene (1,2-$F_2C_6H_4$) and fluorobenzene (C_6H_5F) were stirred over aluminium oxide (Al_2O_3) for two hours, filtered and then stirred over CaH_2 overnight before being vacuum distilled, subsequently degassed by three freeze-pump-thaw cycles and stored over 3 Å molecular sieves. $CoCl_2$ was purchased from Sigma-Aldrich and dried at 160°C for 48 hours prior to use. Norbornadiene (bicyclo[2.2.1]hepta-2,5-diene, abbreviated as NBD) was purchased from Sigma-Aldrich, stirred with freshly cut small pieces of sodium metal for 24 hours and then vacuum distilled , subsequently degassed by three freeze-pump-thaw cycles and store over activated 3 Å molecular sieves. The chelating phosphines 1,2-bis(dicyclohexylphosphino)ethane (dcpe) and 1,4-bis(dicyclohexylphosphino)butane (dcpb) were purchased from Sigma-Aldrich, stored in a MBraun glovebox under an argon atmosphere and used as received. Sodium tetrakis[3,5-bis(trifluoromethyl)phenyl]borate[S1] (Na[BAr^F_4], Ar^F = 3,5-$(CF_3)_2C_6H_3$) was stored in a MBraun glovebox under an argon atmosphere and used as a crystalline solid. High purity hydrogen gas (99.9%) was employed in the solid/gas and solution/gas transformations. All other chemicals were purchased from commercial sources and used as received. Elemental analyses were conducted by Mr Stephan Boyer at London Metropolitan University.

S.1.2. NMR Spectroscopic Data

Solution NMR data were collected on either a Bruker Avance III NMR spectrometer (equipped with a 11.75 T magnet) or a BrukerAvance III HD nanobay NMR spectrometer (equipped with a 9.4 T magnet) at 298 K unless otherwise started. The temperature for Variable Temperature (VT) NMR experiments at low temperatures was externally calibrated to observe the chemical-shift separation between the OH and CH_3 resonances in methanol. Non-deuterated solvents were locked to either standard external neat CD_2Cl_2 or 10% fluorobenzene in C_6D_6 samples. Residual protio solvent resonances were used as a reference for ^{1}H NMR spectra. A small amount of CD_2Cl_2 was added as a reference for ^{2}H NMR spectra. $^{31}P\{^{1}H\}$ NMR spectra were referenced externally to 85% H_3PO_4 (D_2O). $^{19}F\{^{1}H\}$ NMR spectra were referenced externally to a 1% $CFCl_3$ sample in $CHCl_3$. $^{11}B\{^{1}H\}$ NMR spectra were referenced externally to a 5% $BF_3 \cdot OEt_2$ sample in C_6D_6. All chemical shifts (d) are quoted in ppm and coupling constants (J) in Hz. In general, solution ^{19}F and ^{11}B NMR resonances for all the compounds reported are not included since they comprise of a single resonance at d -62.9 and -6.5 respectively, corresponding the $[BAr^{F}_4]^-$ anion. Magnetic susceptibility measurements were performed in CD_2Cl_2 according to the Evans NMR method.[S2]

S.1.3. Mass Spectrometry Data

Electrospray ionization mass spectrometry (ESI-MS) was carried out using a Bruker MicroTOF instrument directly connected to a modified Innovative Technology glovebox. Typical acquisition parameters were used (sample flow rate: 4 μL min^{-1} , nebulizer gas pressure: 0.4 bar, drying gas: argon at 333 K flowing at 4 L min^{-1} , capillary voltage: 4.5 kV, exit voltage: either 15 or 60 V). The spectrometer was calibrated using a mixture of tetraalkyl ammonium bromides $[N(C_nH_{2n+1})_4]Br$ (n = 2-8, 12, 16 and 18) in dichloromethane. Samples were diluted to a concentration of ca. < 1×10^{-6} M in the appropriate solvent (dichloromethane, 1,2-difluorobenzene) before sampling by ESI-MS.

S.2. Synthetic Procedures and Characterisation data

S.2.1. Preparation and Characterisation of [Co(L1)-NBD][BAr^F_4]

One Schlenk flask was charged with $CoCl_2$ (20 mg, 154 mmol) and another with 1,2-bis(dicyclohexylphosphino)ethane (69.5 mg, 154 mmol). Both solids were dissolved in tetrahydrofuran (3 mL each) and the solution containing the phosphine was added dropwise to that of $CoCl_2$ via cannula with vigorous stirring. The resulting deep blue solution was stirred for 10 minutes before being added dropwise to a solution of $NaBAr^F_4$ (136.5 mg, 154 mmol) in tetrahydrofuran (5 mL), which underwent a rapid colour change to a very dark blue solution. Norbornadiene (0.2 mL) was added to the resultant solution was stirred at room temperature overnight.

One J Youngs flask was charged with KC_8 (50 mg, 377 mmol) and suspended in tetrahydrofuran (3 mL). The deep blue solution was transferred onto the KC_8 suspension at -78°C and then allowed to warm to room temperature and stirred for 2 hours. The remaining graphite was removed by filtration of the resultant green solution and the solvent removed in vacuo. The resultant green solid was dissolved in dichloromethane (5 mL) and stirred for 5 minutes. *n*-pentane (50 mL) was added to give a lilac suspension which was filtered via cannula. The solid was washed with *n*-pentane (3 x 5 mL), and dried *in vacuo* (< 5 x 10^{-2}, 45 min), to yield a lilac powder [Co(**L1**)-NBD][BAr^F_4]] (140 mg, 100 mmol 65 % yield). Crystals suitable for X-ray diffraction study were obtained by slow diffusion of *n*-pentane (25 mL) into a solution of the lilac solid (140mg) in dichloromethane (1 ml) in a crystallisation tube fitted with a J Young valve at room temperature. To give lilac block like crystals (100 mg, 69 mg, 45 % yield).

^{1}H NMR (400 MHz, CD_2Cl_2, 298 K): d 7.72 (m, 13 H, ortho-BAr^F_4), 7.56 (s, 7 H, para-BAr^F_4), 4.26 (s, 4H, NBD-**H**C=CH) 3.91 (s, 2H, NBD-**H**C-HC=CH), 2.40 (br, d, 4H, Cy C**H**-P) 2.22 (br, t, 4H, backbone C**H**$_2$), 1.95-1.60 (br, m, 24H, overlapping aliphatic CH), 1.55 (s, 2H, NBD-**H**$_2$C) 1.5-1.15 (br, m, 24H, overlapping aliphatic CH)

^{31}P{^{1}H} NMR (162 MHz, CD_2Cl_2, 298 K): δ 61.0 (br, s)

Magnetic measurement using Evans method of a CD_2Cl_2 sample: μ_{eff}(298 K) = 1.5(1) µB.

Magnetic measurement using SQUID magnetometer methods: 2.16 µB.

Elemental analysis found (calculated) for C65 H68 B1 F24 P2 Co: C, 54.22 (54.33); H, 4.56(4.77)%.

ESI-MS m/z: no parent-ion observed.

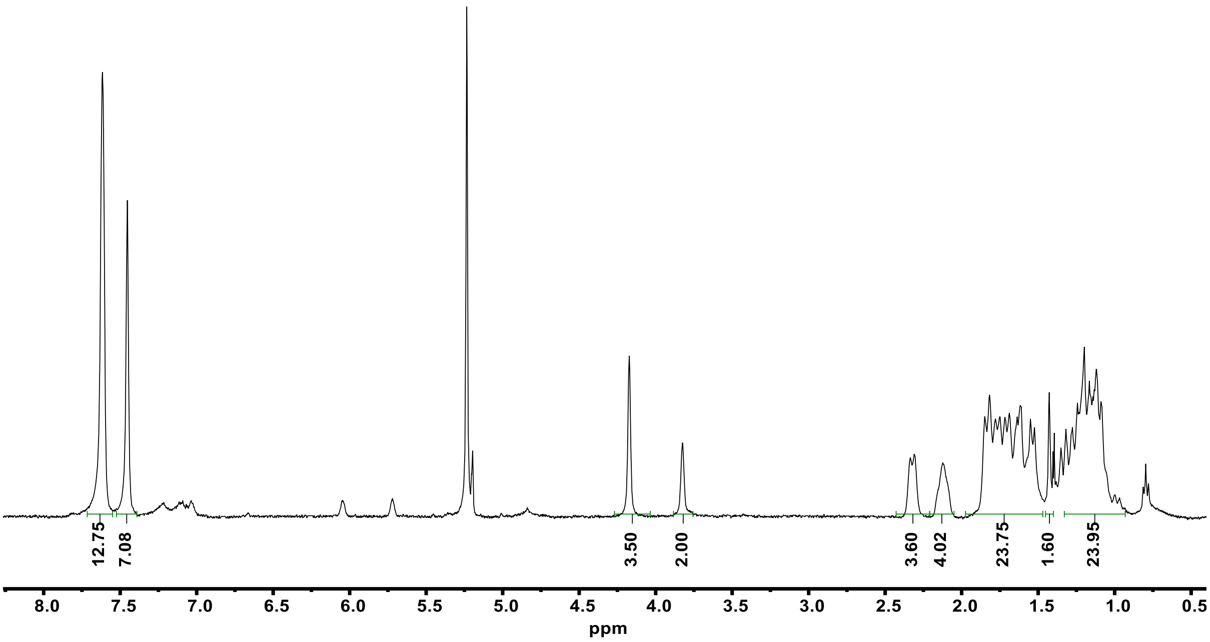

Figure S1. The solution ^{1}H NMR (CD_2Cl_2, 298 K, 400 MHz) spectrum of [Co(**L1**)-NBD][$BAr^F{}_4$]

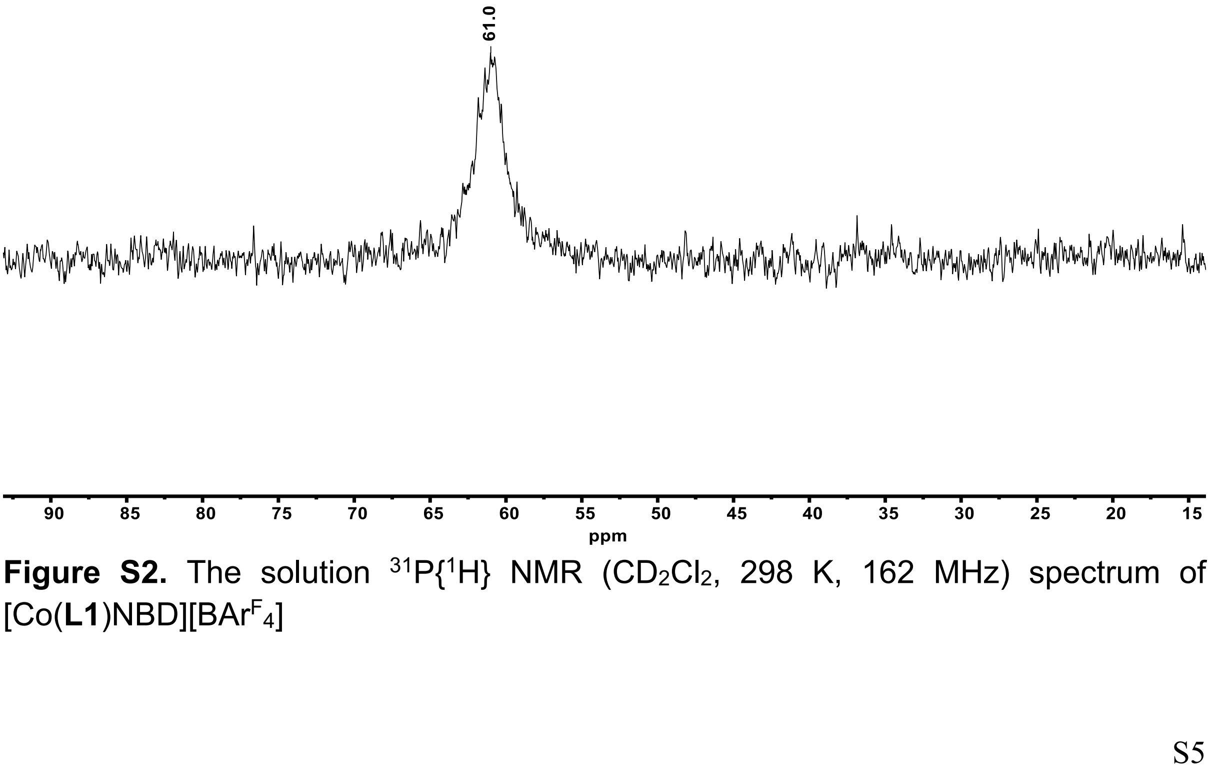

Figure S2. The solution $^{31}P\{^1H\}$ NMR (CD_2Cl_2, 298 K, 162 MHz) spectrum of [Co(**L1**)NBD][$BAr^F{}_4$]

S.2.2. Preparation and Characterisation of [Co(L1)(η-FC_6H_5)][BAr^F_4]

One J Youngs flask was charged with [Co(**L1**)-NBD][BAr^F_4] (80 mg, 56 mmol) and dissolved in fluorobenzene (2 ml). The solution was freeze-pump-thaw degrassed three times and refilled with H_2 (2 atm), the solution rapidly changed from lilac to yellow. After stirring for 20 minutes the solution was freeze-pump-thaw degassed three times and refilled with Ar (1 atm). The solution was transferred via canula to a crystallisation tube fitted with a J Young valve and crystals suitable for X-ray diffraction were obtained by slow diffusion of *n*-pentane (25 mL) into this solution at room temperature. This yielded brown plates of [Co(**L1**)(η-FC_6H_5)][BAr^F_4] (75 mg, 52 mmol, 90 % yield)

^{1}H NMR (400 MHz, CD_2Cl_2): δ 7.77 (m, 8 H, ortho-BAr^F_4), 7.61 (m, 4 H, para-BAr^F_4), 6.29 (s, 2 H, o-C_6H_5F), 6.15 (s, 2 H, m-C_6H_5F), 5.73 (s, 1 H, p-C_6H_5F) 1.99 (t, 8 H) 1.90 (s, 2 H), 1.84 (br d, 2 H), 1.69 (br, 14 H), 1.34 (br, 18 H), 1.12 (q, 4 H).

^{31}P{^{1}H} NMR (162 MHz, CD_2Cl_2, 298 K): *δ* 95.5 (s)

^{19}F NMR (376 MHz, THF-d8, 23°C): δ -63.43 (s, 24 F, B[(3,5-(CF3)2)C6H3]4), -163.25 (s, 1 F, C_6H_5F)

Magnetic measurement using Evans method of a CD_2Cl_2 sample: μ_{eff}(298 K) = 1.1(1) μB.

Magnetic measurement using SQUID magnetometer methods: 1.61 μB.

Elemental analysis found (calculated) for C64 H65 B1 F25 P2 Co: C, 53.22 (53.35); H, 4.46 (4.55)%.

ESI-MS m/z found (calculated) for C32 H53 Co F P2 $[M]^+$: 577.2937 (577.2933)

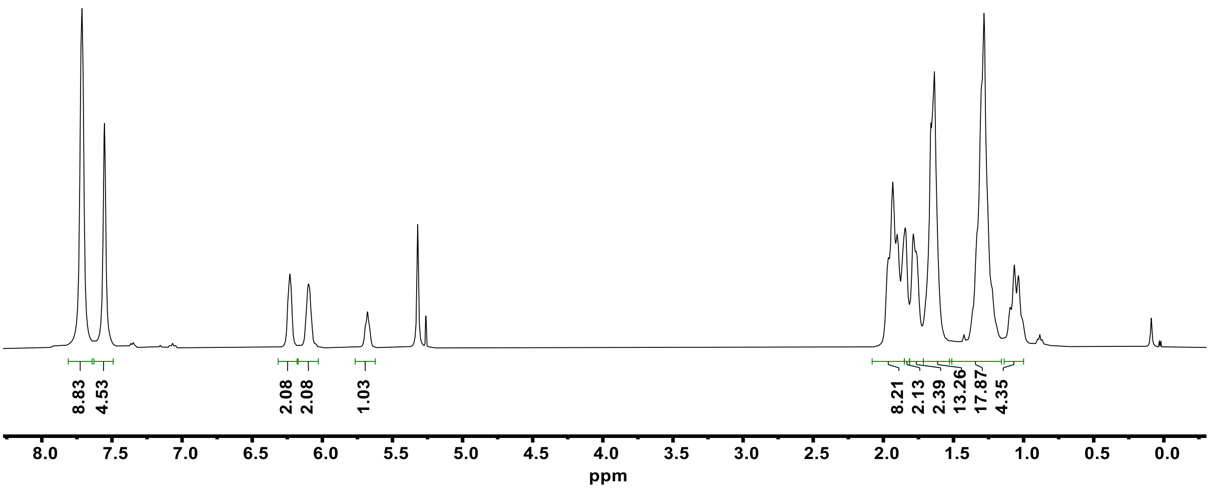

Figure S3. The solution ^{1}H NMR (CD_2Cl_2, 298 K, 400 MHz) spectrum of [Co(**L1**)(η-FC_6H_5)][$BAr^F{}_4$]

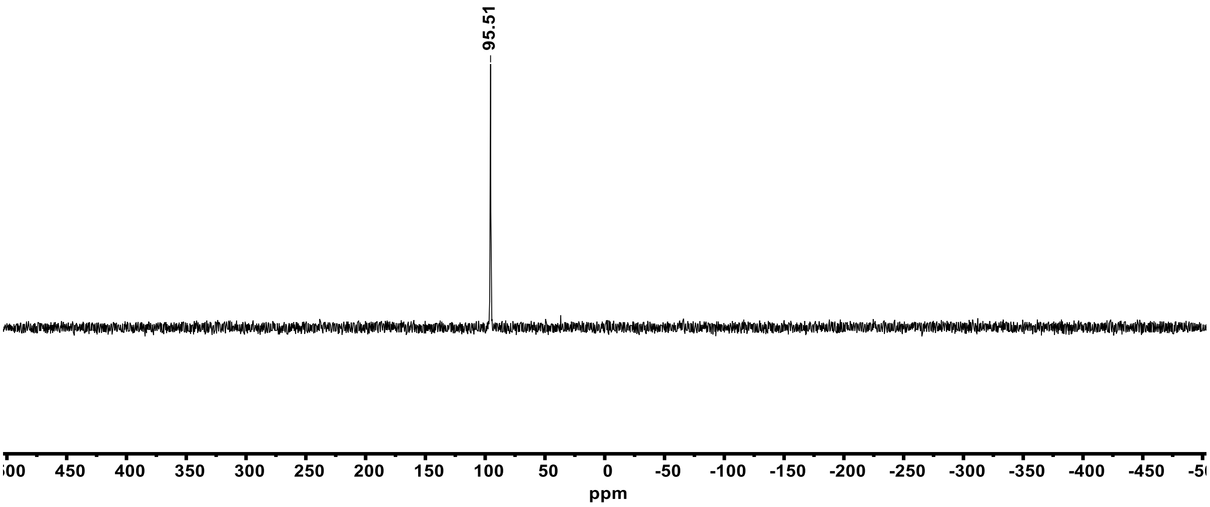

Figure S4. The solution $^{31}P\{^{1}H\}$ NMR (CD_2Cl_2, 298 K, 162 MHz) spectrum of [Co(**L1**)(η-FC_6H_5)][$BAr^F{}_4$]

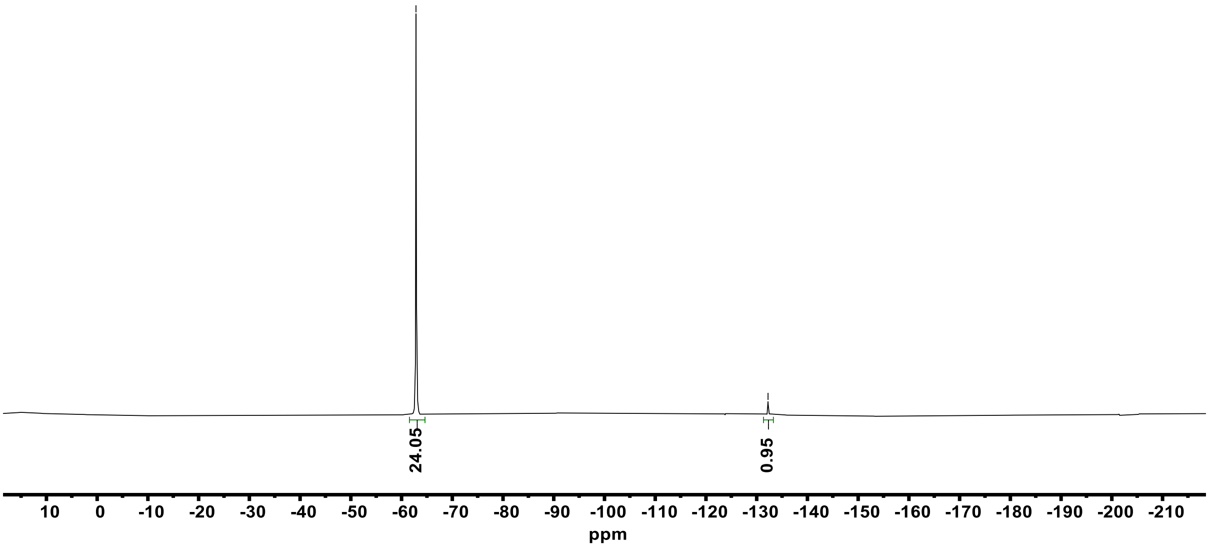

Figure S5. The solution $^{19}F\{^{1}H\}$ NMR (CD_2Cl_2, 298 K, 377 MHz) spectrum of $[Co(\mathbf{L1})(\eta\text{-}FC_6H_5)][BAr^F_4]$

S.2.3. Preparation and Characterisation of [Co(L2)-NBD][BAr^F_4]

One J Youngs flask was charged with $CoCl_2$ (20 mg, 154 mmol) and another with 1,2-bis(dicyclohexylphosphino)butane (69.5 mg, 154 mmol). Both solids were dissolved tetrahydrofuran (3 mL each) and the solution containing the phosphine was added dropwise to that of $CoCl_2$ via cannula with vigorous stirring. The resulting deep blue solution was stirred for 10 minutes before being added dropwise to a solution of $NaBAr^F_4$ (136.5 mg, 154 mmol) in tetrahydrofuran (5 mL). Norbornadiene (0.2 mL) was added to the resultant deep blue solution which was stirred at room temperature overnight.

One J Youngs flask was charged with KC_8 (50 mg, 377 mmol) and suspended in tetrahydrofuran (3 mL). The deep blue solution was transferred onto the KC_8 suspension at -78°C and then allowed to warm to room temperature and stirred for 2 hours. The remaining graphite was removed by filtration of the resultant green solution and the solvent removed in vacuo. The resultant green solid was dissolved in dichloromethane (5 mL) at -78°C and stirred for 5 minutes. *n*-pentane (50 mL) was added to give a dark green suspension which was filtered via cannula. The solid was washed with *n*-pentane (3 x 5 mL), and dried *in vacuo* (< 5 x 10^{-2}, 45 min), to yield a dark green powder. Crystals of [Co(**L2**)-NBD][BAr^F_4] (70 mg, 48 mmol, 31 % yield) suitable for an X-ray diffraction study were obtained by slow diffusion of *n*-pentane (25 mL) into a solution of the green solid in 1,2-difluorobenzene (1 ml).

This procedure was only successful on the provision that all solvents were freshly freeze-pump-thaw degassed and manipulations were carried out with extreme care.

^{1}H NMR (400 MHz, d_8-THF): δ 7.71 (m, 8 H, ortho-BAr^F_4), 7.50 (m, 4 H, para-BAr^F_4) cation is NMR silent.

$^{31}P\{^{1}H\}$ NMR: (162 MHz, d_8-THF, 298 K): silent

Magnetic measurement using Evans method of a CD_2Cl_2 sample: μ_{eff}(298 K) 3.2(2) μB.

Magnetic measurement using SQUID magnetometer methods: 3.14 μB

Elemental analysis found (calculated) for C67 H72 B F24 P2 Co: C, 54.89 (54.78); H, 5.08 (5.21)%.

ESI-MS m/z: no parent-ion observed.

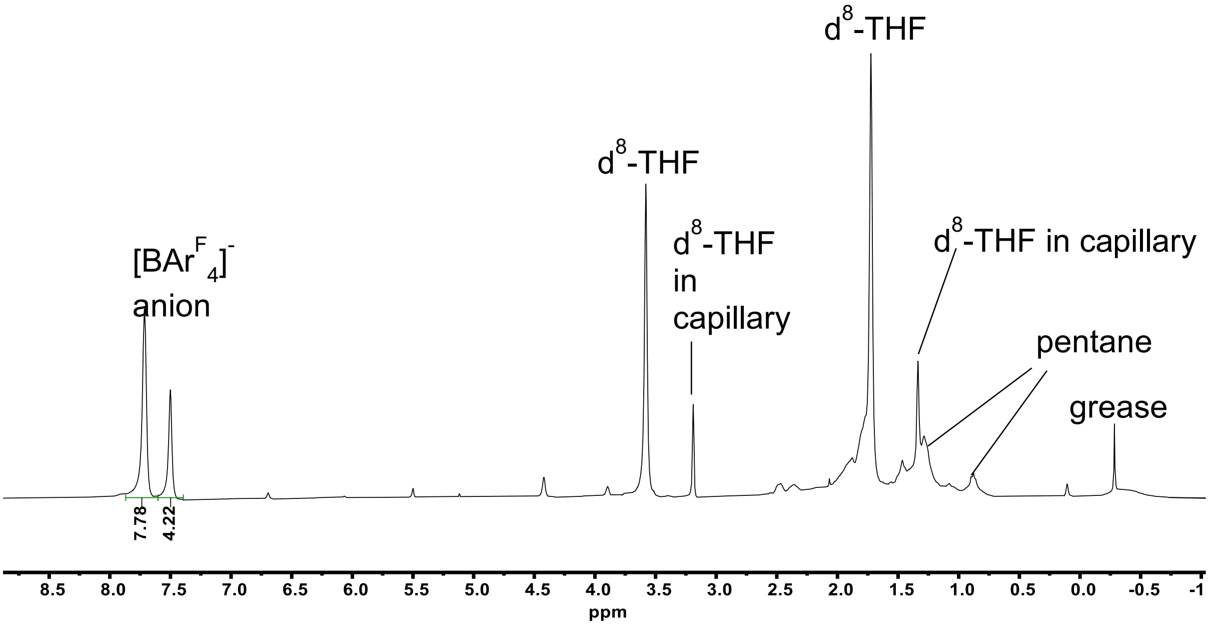

Figure S6. The solution ^{1}H NMR (CD_2Cl_2, 298 K, 400 MHz) spectrum of [Co(**L2**)-NBD][BAr$^F_4$]. Collected under Evan's measurement conditions.

S.2.4. Preparation and Characterisation of [Co(L2)-NBA][BAr$^F_4$]

For single crystal x-ray studies

Crystals of [Co(**L2**)-NBD][BAr$^F_4$] (10 mg) were placed in a J Youngs NMR tube and exposed to a hydrogen atmosphere (2 bar) for 1 hr at room temperature, after which the crystals were rapidly transferred to the diffractometer and a structure of the [Co(**L2**)-NBA][BAr$^F_4$] could be collected.

For solid state NMR studies

Crystals of [Co(**L2**)-NBD][BAr$^F_4$] (45 mg) were packed in an uncapped solid state NMR rotor, inside an argon filled glove box. The rotor was then placed in a custom built glass J Young flask[S3] and exposed to a hydrogen atmosphere (2 bar) for 1 hr at room temperature, after this time the rotor was capped and the sample immediately transferred to the NMR spectrometer.

For SQUID magnetometer studies

Crystals of [Co(**L2**)-NBD][BAr$^F_4$] were placed within a Spectrosil® sample holder, shown in Figure S14, this was exposed to a hydrogen atmosphere (2 bar) for 1 hr, before sealing under vacuum and rapid transfer of the sample to the SQUID magnetometer. Magnetic measurement using SQUID magnetometer methods: 3.38 μB

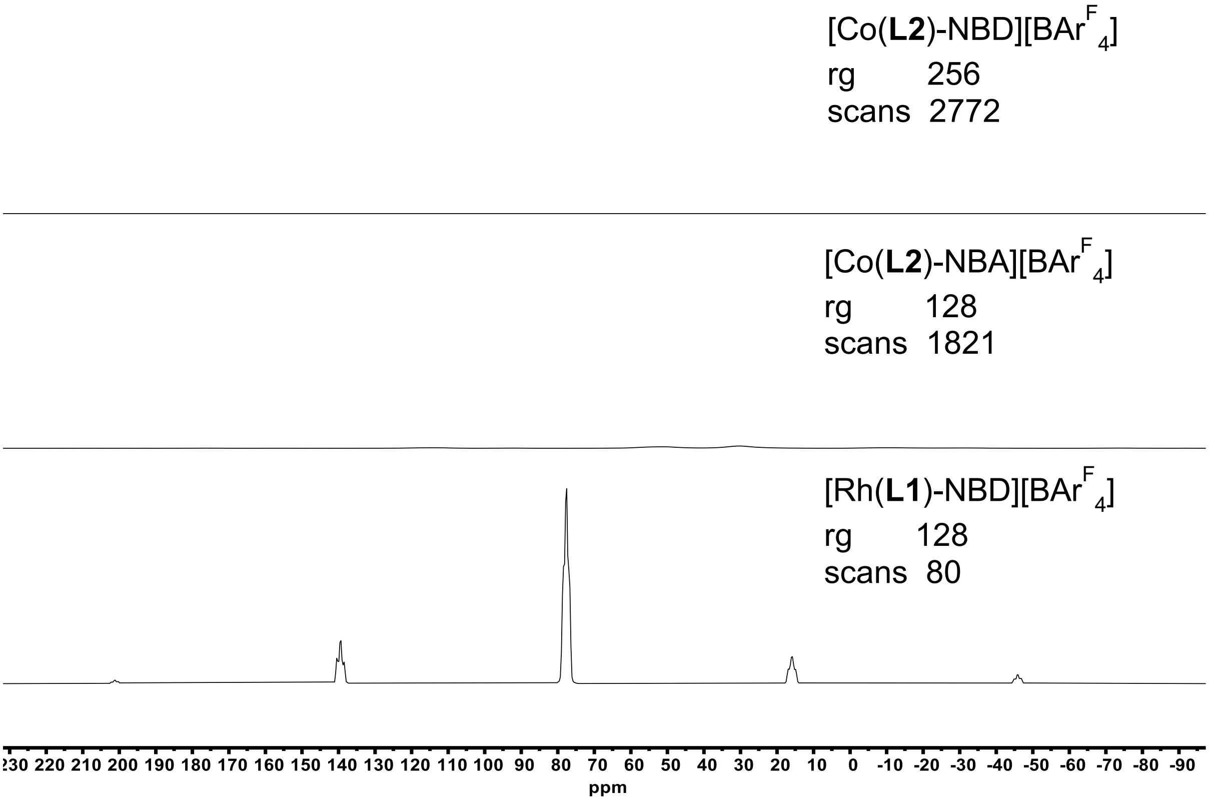

Figure S7. The $^{31}P\{^{1}H\}$ SSNMR (162 MHz, 298 K, 10 kHz spin rate) spectrum of [Co(**L2**)-NBD][BAr^{F}_{4}], [Co(**L2**)-NBA][BAr^{F}_{4}] and [Rh(**L1**)-NBD][BAr^{F}_{4}]. Normalised for receiver gain and number of scans. Showing both cobalt complexes giving silent $^{31}P\{^{1}H\}$ SSNMR spectrum. Receiver gain is abbreviated to rg.

S.3. Crystallographic and Refinement Data

S.3.1. Crystal Structure Determinations

Single crystal X-ray diffraction data for all samples were collected as follows: a typical crystal was mounted on a MiTeGen Micromount using perfluoropolyether oil and cooled rapidly to the collection temperature in a stream of nitrogen gas using an Oxford Cryosystems Cryostream unit.[S4] The structures of [Co(**L1**)-NBD][BAr^F_4] and [Co(**L1**)(η-FC_6H_5)][BAr^F_4] were collected at the Oxford Chemical Crystallography Service from the University of Oxford, with an Agilent SuperNova diffractometer (Cu Kα radiation, λ = 1.54180 Å). [Co(**L2**)-NBD][BAr^F_4] and [Co(**L2**)-NBA][BAr^F_4] were collected at the National Crystallography Service, University of Southampton, Southampton, UK, using a Rigaku 007HF diffractometer equipped with Varimax confocal mirrors (Cu Kα radiation, λ = 1.54178 Å), an AFC11 goniometer, and HyPix 6000 detector.

Raw frame data were reduced using CrysAlisPro.[S5] The structures were solved using SHELXT[S6] and refined using full-matrix least squares refinement on all F^2 data using the SHELXL-2018[S7] using the interface OLEX2.[S8] All hydrogen atoms were placed in calculated positions (riding model).

S.3.2. Additional Comments Crystal Structures and Refinement Data

[Co(L1)–NBD][BAr^F_4]

Four -CF_3 groups are disordered (ca. 56:44, 56:44, 53:47 51:49) and 1,2 and 1,3 equal distance geometrical and displacement parameter restraints were applied to each pair of disorder components.

[Co(L1)(η–FC_6H_5)][BAr^F_4]

Four -CF_3 groups are disordered (ca. 74:26, 69:31, 62:38 60:40) and 1,2 and 1,3 equal distance geometrical and displacement parameter restraints were applied to each pair of disorder components. The coordinated fluorobenzene is disordered over three positions (ca. 52:32:16) and 1,2 equal distance geometrical and displacement parameter restraints were applied to all of the disorder components.

[Co(L2)–NBD][BAr$^F{}_4$]

The [BAr$^F{}_4$]$^-$ anion displays whole molecule disorder (ca. 52:48) and 1,2 and 1,3 equal distance geometrical restraints were applied to both components. Displacement parameter restraints were applied to the whole structure. One cyclohexane moiety in the asymmetric unit is disordered (ca. 65:35) and 1,2 and 1,3 equal distance geometrical restraints were applied to both components. The C4 backbone was disordered via crystallographic symmetry over two positions (50:50). Displacement parameter restraints and 1,2 and 1,3 equal distance geometrical restraints between equivalent pairs of atoms were applied to coordinated NBD.

[Co(L2)–NBA][BAr$^F{}_4$]

A 1,2 equal distance geometrical restraint was applied between all secondary and tertiary C-atoms of NBA. 1,2 and 1,3 equal distance geometrical restraints were applied between all secondary C-atoms of NBA. A 1,2 equal distance geometrical restraint was applied to the disordered C4 backbone. 1,2 and 1,3 equal distance restraints were applied to the disorder components of the [BAr$^F{}_4$]$^-$ anion.

	[Co(**L1**)–NBD][BAr^F_4]	[Co(**L1**)(η–FC_6H_5)][BAr^F_4]	[Co(**L2**)–NBD][BAr^F_4]	[Co(**L2**)–NBA][BAr^F_4]
Chemical formula	$C_{65}H_{68}BCoF_2P_2$	$C_{64}H_{65}BCoF_{25}P_2$	$C_{67}H_{72}BCoF_{24}P_2$	$C_{67}H_{76}BCoF_{24}P_2$
Formula weight	1436.87	1440.84	1464.92	1468.95
Temperature (K)	150	150	100	100
Crystal system	triclinic	triclinic	monoclinic	monoclinic
Space group	P-1	P-1	C2/c	C2/c
a (Å)	12.8234(9)	12.7272(5)	19.3071(2)	19.0831(3)
b (Å)	12.9744(8)	12.8527(5)	17.1586(3)	17.7975(3)
c (Å)	19.8379(11)	20.1804(7)	20.3552(2)	20.2129(3)
α (deg)	92.463(5)	91.965(3)	90	90
β (deg)	90.162(5)	90.053(3)	93.4560(10)	92.9714(12)
γ (deg)	96.188(5)	97.304(4)	90	90
Volume ($Å^3$)	3278.2(4)	3272.3(2)	6731.06(15)	6855.70(17)
Z	2	2	4	4
ρ_{calc} g/cm^3	1.456	1.462	1.446	1.423
μ/mm^{-1}	3.480	3.511	3.401	3.339
Reflections collected	24258	37212	38436	38479
Independent reflections	12435	37212	6276	6390
Restraints / parameters	537 / 957	1420 / 1080	1851 / 756	579 / 577
R_{int}	0.0880		0.0338	0.0456
R_1 [I > 2σ(I)]	0.0931	0.1024	0.0609	0.0796
wR_2 [all data]	0.2816	0.3072	0.1772	0.2144
GooF	1.018	1.058	1.048	1.034
Residual electron density (e Å^{-3})	1.22 / -0.56	1.52 / -0.65	0.82 / -0.58	0.92 / -0.48
CCDC no.	1967463	1967464	1967534	1967533

Table S1: Selected crystallographic and refinement data.

S.3.3. Single Crystal X-ray Diffraction Images

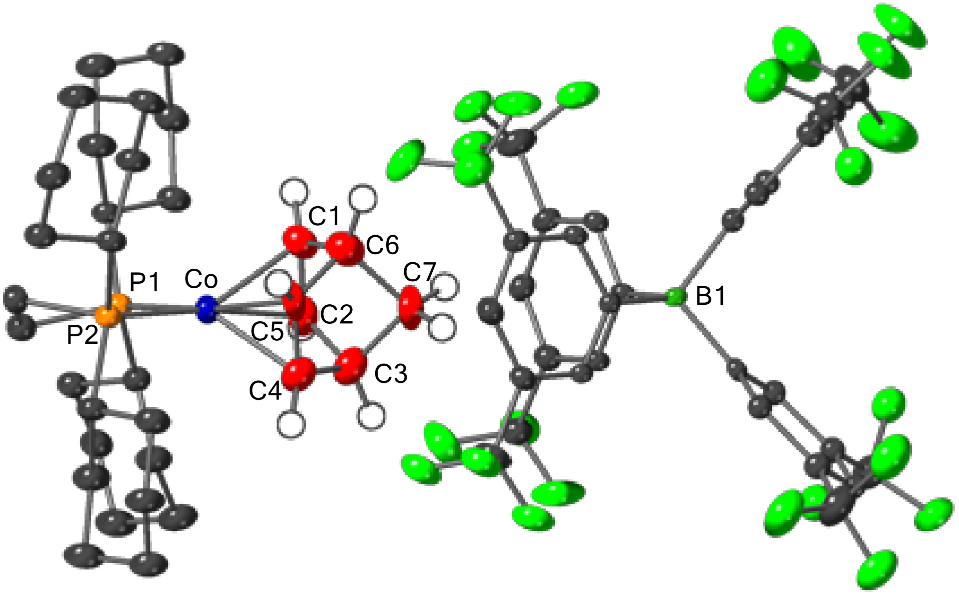

Figure S8. Molecular structure of [Co(**L1**)-NBD][BAr^F$_4$]. Thermal ellipsoids set at 30 % and hydrogen atoms from the phosphine ligand and [BAr^F$_4$]$^-$ anion removed for clarity. Only the major disorder component of [BAr^F$_4$]$^-$ anion shown.

Selected bond lengths (Å) and angles (°): Co1-P1 2.226(2), Co1-P2 2.2298(19), Co1-C5 2.073(7), Co1-C2 2.091(8), Co1-C4 2.092(9), C1-C2 1.410(12), C5-C4 1.403(12), P1-Co-P2 87.48(7), CoP1P2/Co(centC1C2)(centC4C5) 22.0(2).

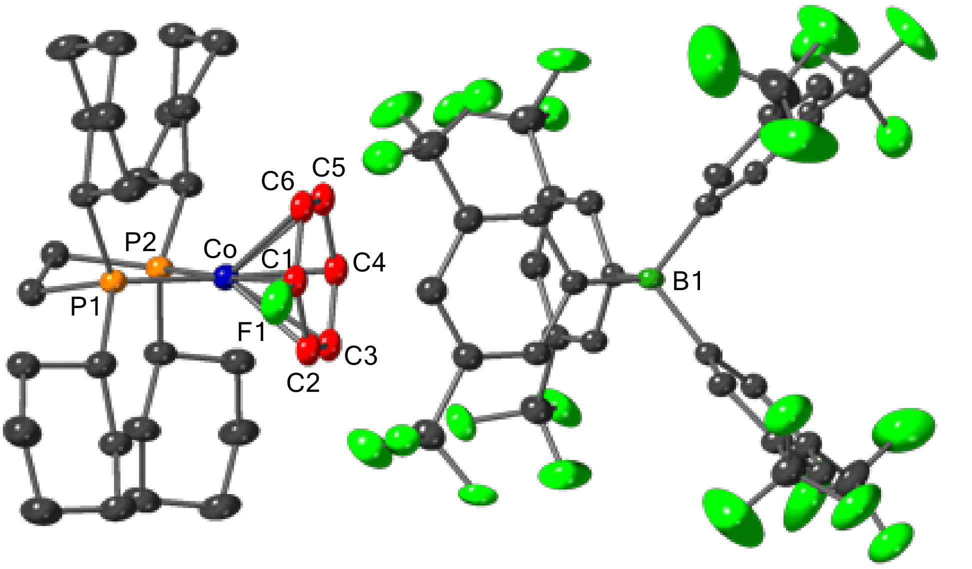

Figure S9. Molecular structure of [Co(**L1**)(η-FC_6H_5][BAr^F$_4$]. Thermal ellipsoids set at 30 % and hydrogen atoms removed for clarity. Only the major disorder component of [BAr^F$_4$]$^-$ anion shown.

Selected bond lengths (Å) and angles (°): Co1-P1 2.1839(16), Co1-P2 2.1803(17), Co-centC1C2C3C4C5C6 1.601(5), P1-Co-P2 86.69(6).

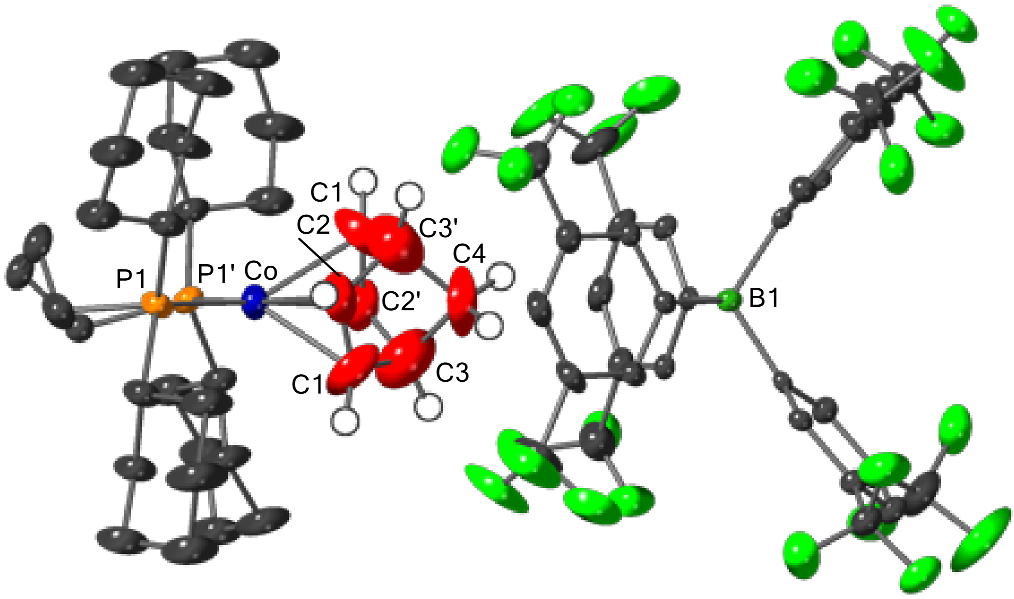

Figure S10. Molecular structure of [Co(**L2**)-NBD][BAr$^F{}_4$]. Thermal ellipsoids set at 30 % and hydrogen atoms from the phosphine ligand and [BAr$^F{}_4$]$^-$ anion removed for clarity. Only the major disorder component of [BAr$^F{}_4$]$^-$ anion shown.

Selected bond lengths (Å) and angles (°): Co1-P1 2.2717(8), Co1-C1 2.212(6), Co1-C2 2.110(4), P1-Co-P2 103.09(4), CoP1P2/Co(centC1C2)(centC1'C2') 28.0(3).

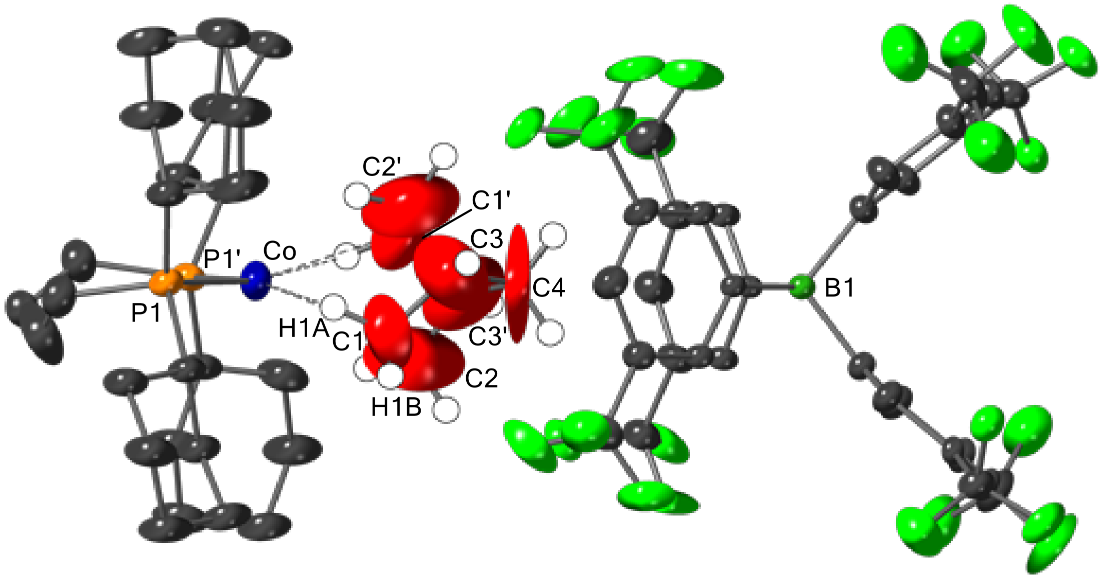

Figure S11. Molecular structure of [Co(**L2**)-NBA][BAr$^F{}_4$]. Thermal ellipsoids set at 30 % and hydrogen atoms from the phosphine ligand and [BAr$^F{}_4$]$^-$ anion removed for clarity. Only the major disorder component of [BAr$^F{}_4$]$^-$ anion shown.

Selected bond lengths (Å) and angles (°): Co1-P1 2.232(1), Co1-C1 2.612(16), P1-Co-P2 108.94(7), CoP1P1'/CoC1C1' 39.9(8).

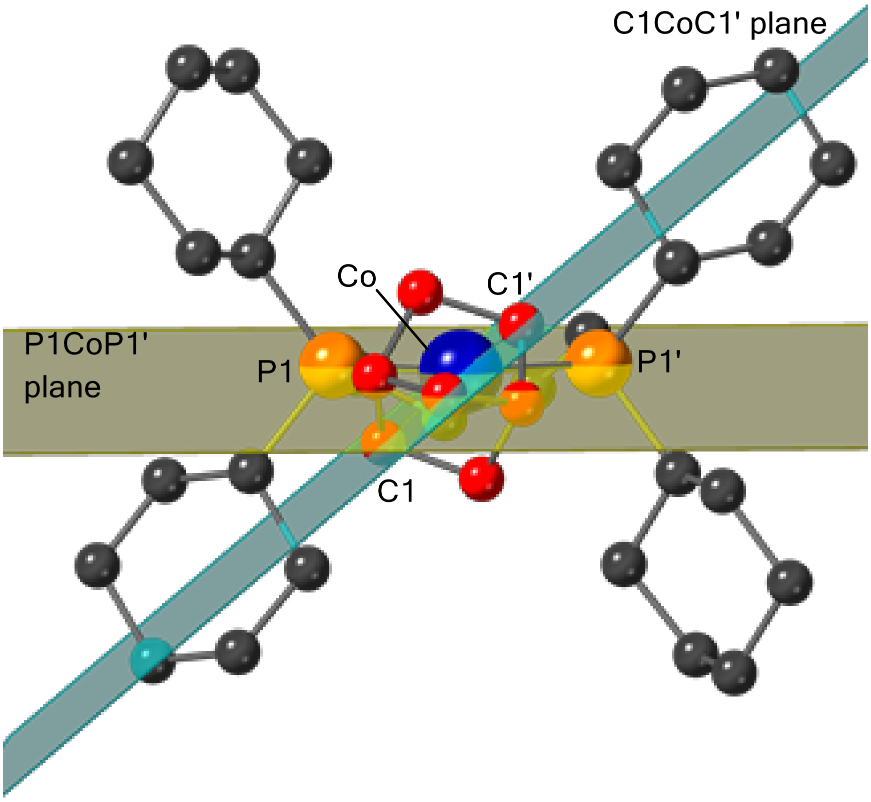

Figure S12. Molecular structure of [Co(**L2**)-NBA][BAr$^F{}_4$]. Ball and stick representation used for clarity. The yellow plane is the P1CoP1' plane and the blue plane is the C1CoC1' plane. The angle between these is used as a measure of how twisted away from square planar the NBA fragment is.

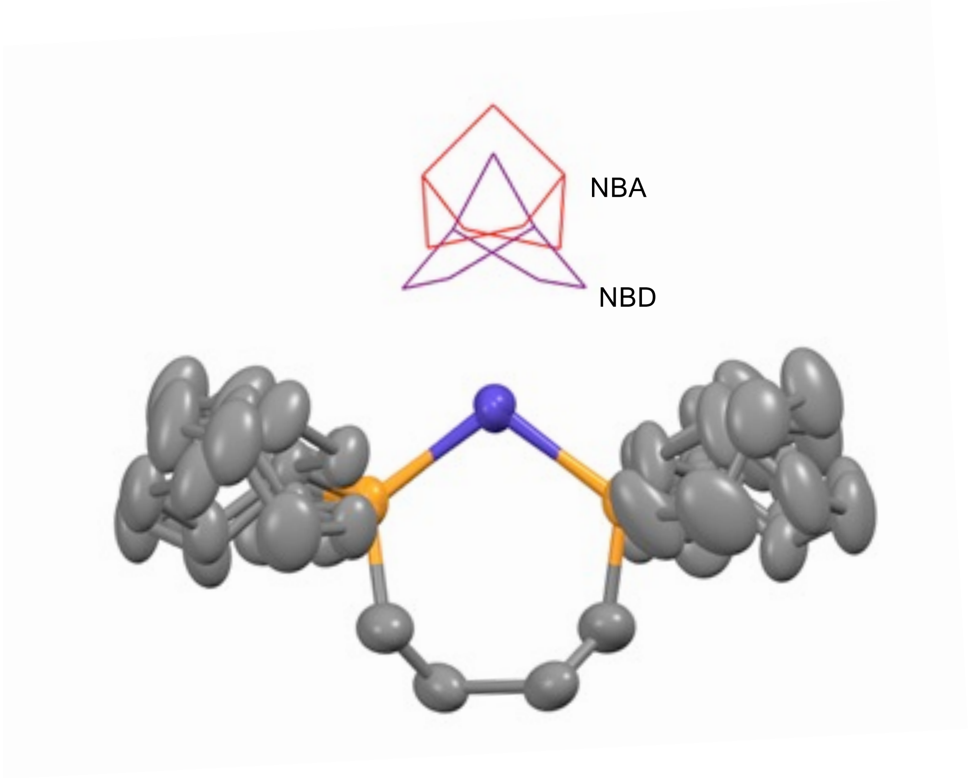

Figure S13. Overlay of the structures of [Co(**L2**)-NBA][BAr$^F{}_4$] and [Co(**L2**)-NBD][BAr$^F{}_4$]. Thermal ellipsoids set at 50 % and the [BAr$^F{}_4$]$^-$ anions, hydrogens and disordered components were removed for clarity. NBA (red) and NBD (purple) are displayed as a wireframe for clarity.

S.4. Magnetic Characterisation

Zero-field cooled magnetization data were collected as a function of temperature from samples sealed under vacuum within Spectrosil® sample holders, shown in Figure S14, in an applied field of 1000 Oe using an MPMS 5 Quantum Design SQUID magnetometer.

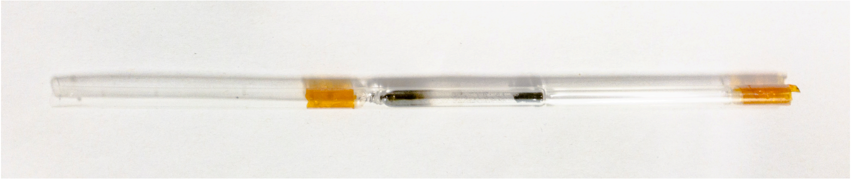

Figure S13. Picture of sample holder

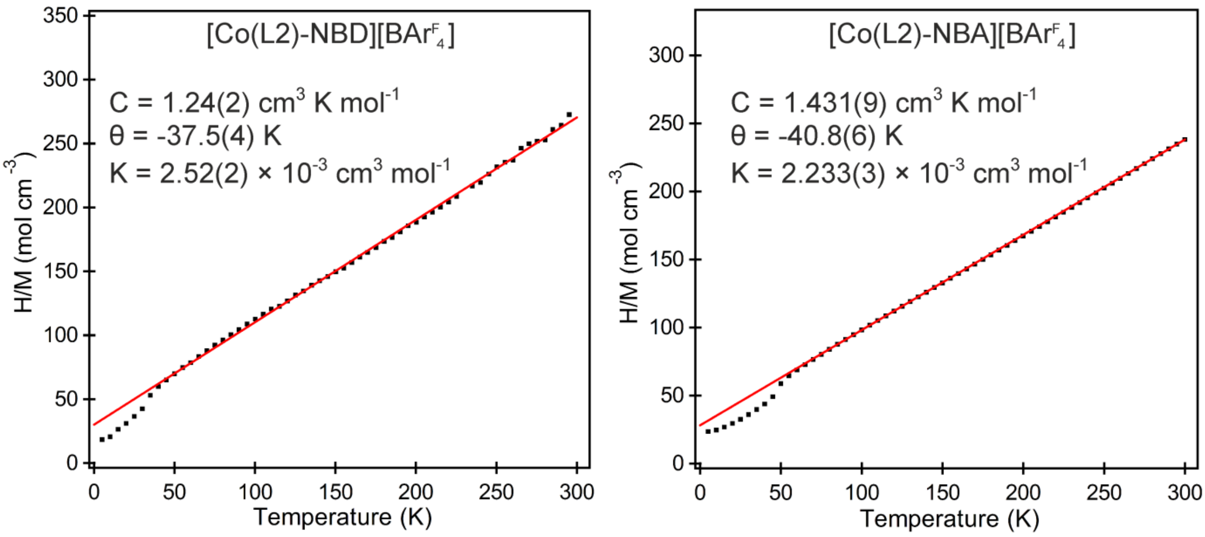

Figure S14. Cure-Weiss fits to the magnetisation data collected from [Co(**L2**)–NBD][$BAr^F{}_4$] and [Co(**L2**)–NBA][$BAr^F{}_4$] in the temperature range 50 < T/K < 300.

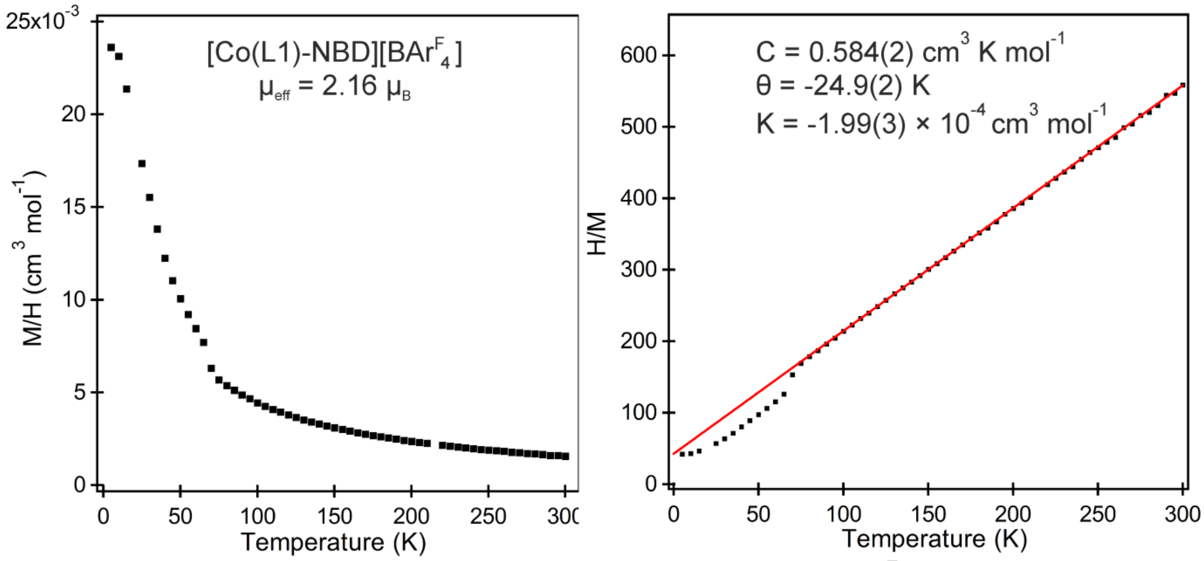

Figure S15. Magnetisation data (left) from [Co(**L1**)–NBD][BAr^F_4] and Curie-Weiss fit (right) to these data over the temperature range 75 < T/K < 300.

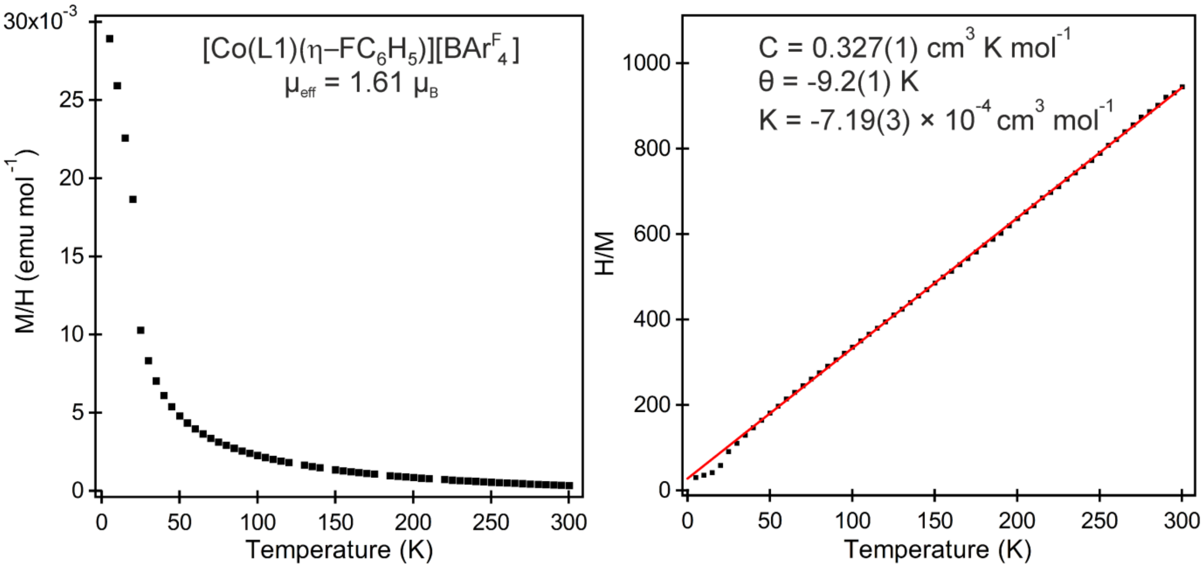

Figure S16. Magnetisation data (left) from [Co(**L1**)(η–FC_6H_5)][BAr^F_4] and Curie-Weiss fit (right) to these data over the temperature range 50 < T/K < 300.

The magnetisation data collected from both from [Co(**L1**)–NBD][BAr^F_4] and [Co(**L1**)(η–FC_6H_5)][BAr^F_4] can be fitted to the Curie Weiss law ($\chi = C/(T-\theta) + K$) over the temperature range 75 < T/K < 300 to yield Curie constants of 0.584(2) cm^3 K mol^{-1} and 0.327(1) cm^3 K mol^{-1} respectively, giving calculated μ_{eff} values of 2.16 μ_B and 1.61 μ_B respectively. These values are intermediate between those expected for a ^{1}Co spin-state (S = 0, C = 0 cm^3 K mol^{-1}, μ_{eff} = 0 μ_B) and a ^{3}Co spin-state (S = 1, C = 1 cm^3 K mol^{-1}, μ_{eff} = 2.82 μ_B). This suggests that these complexes are tending to a ^{1}Co spin-state in the 75 < T/K < 300 temperature range, but that there is still significant mixing with a ^{3}Co state, as indicated by the non-zero values for the susceptibilities.

S.5. Computational Methods

S.5.1. Solid State Calculations

All static Kohn-Sham DFT calculations were performed on periodic models of the studied cobalt complexes, employing the Gaussian Plane Wave (GPW) formalism as implemented in the QUICKSTEP[S9] module within the CP2K program suite (Version 5.0).[S10] Molecularly optimized basis sets of double-ζ quality plus polarization in their short-range variant (DZVP-MOLOPT-SR-GTH)[S11] were used on all atomic species. The interaction between the core electrons and the valence shell (Co: 17, B: 3, C: 4, P: 5, F: 7, H: 1 electrons) was described by Goedecker-Teter-Hutter (GTH) pseudo potentials.[S12-14] The generalized gradient approximation (GGA) to the exchange-correlation functional according to Perdew-Burke-Ernzerhof (PBE)[S15-16] was used in combination with Grimme's D3-correction for dispersion interactions.[S17] The auxiliary plane wave basis set was truncated at a cutoff of 500 Ry. The maximum force convergence criterion was set to 10^{-4} Eh·Bohr^{-1}, whilst default values were used for the remaining criteria. The convergence criterion for the self-consistent field (SCF) accuracy was set to 10^{-7} Eh for the geometry optimization. The pressure tolerance for the cell optimizations was set to the default, 100 bar.

The Brillouin zone was sampled using the Γ-point. Initial coordinates for the [Co(**L2**)–NBD][BAr$^F_4$] and [Co(**L2**)–NBA][BAr$^F_4$] structures were obtained from the experimental crystallographic data, with the hydrogen positions normalised where possible with Mercury.[S18] Periodic boundary conditions (PBC) were applied throughout. All geometries were first partially relaxed, keeping the heavy atoms (non-H, F) fixed and the unit cell parameters constant, then fully relaxed without imposing any constraints, including allowing the unit cell parameters to relax. For [Co(**L2**)–NBA][BAr$^F_4$] the C_4 phosphine backbone was relaxed under local C_2 symmetry to account for the disorder present in the structure.

Upon relaxing the [Co(**L2**)–NBA][BAr$^F_4$] unit cell, the alkane retains its overall 1,4-binding motif when optimised as a triplet and the unit cell expands slightly, as seen in Table S2. When optimised as a singlet it reverts to a square planar geometry similar to its Rh analogue.[S19] We were not able to converge the geometry keeping the cell parameters fixed. Upon relaxing the [Co(**L2**)–NBD][BAr$^F_4$] unit cell, the alkene retains its overall binding motif when optimised both as a singlet and as a triplet, with a small expansion of the unit cell volume in both cases.

All computed structures are available as a separate file of Cartesian coordinates.

Table S2. Computed unit cell parameters (Å, °) for [Co(**L2**)–NBA][BAr$^F{}_4$] and [Co(**L2**)–NBD][BAr$^F{}_4$] in the singlet and triplet states, compared with experimental data.[a] Root mean square deviations relative to the experimental structure for all atoms and, in parenthesis, for non-H atoms.

[Co(**L2**)–NBA][BAr$^F{}_4$]								
**Metric**	**a**	**b**	**c**	**α**	**β**	**γ**	**Vol (±%)**	**RMSD[a]**
Exp.	19.0831(3)	17.7975(3)	20.2129(3)	90	92.9714(12)	90	6855.70(17)	-
Singlet PBE-D3	19.2590	17.7980	20.3020	89.7960	92.7750	90.0670	6950.7432 (+1.4)	0.35 (0.22)
Triplet PBE-D3	19.2160	18.0340	20.5720	90.0470	93.1940	89.9820	7117.9718 (+3.8)	0.34 (0.23)
[Co(**L2**)NBD][BAr$^F{}_4$]								
**Metric**	**a**	**b**	**c**	**α**	**β**	**γ**	**Vol (±%)**	**RMSD[a]**
Exp.	19.3071(2)	17.1586(3)	20.3552(2)	90	93.4560(10)	90	6731.06(15)	-
Singlet PBE-D3	19.3970	17.6770	20.4670	89.9950	93.3500	89.9910	7005.7482 (+4.1)	0.22 (0.20)
Triplet PBE-D3	19.3490	17.6730	20.5150	90.0000	93.1660	90.0000	7004.4973 (+4.1)	0.23 (0.19)

The spin energetics were computed on the heavy atom fixed geometries using the range-separated hybrid functional PBE0-TC-LRC,[S20-22] with the auxiliary density matrix method (ADMM)[S23] used to compute the exchange, using the FIT12 auxiliary basis set for Co and the FIT3 auxiliary basis set for all other species. The Coulomb interaction potential was truncated at 5 Å for all systems. The cut-off was tested for 4 Å and 6 Å with similar singlet-triplet gaps, as shown in Table S3.

Table S3. Computed Energies for [Co(**L2**)–NBA][BAr$^F_4$] and [Co(**L2**)–NBD][BAr$^F_4$] in the singlet and triplet states, using the PBE-D3 for both the heavy atom fixed and fully optimised geometries, and using PBE0-TC-LRC-D3 for the heavy atom fixed geometries with different cut-offs R_C.

[Co(**L2**)–NBA][BAr$^F_4$]			
**Functional**	**Singlet SCF Energy (au)**	**Triplet SCF Energy (au)**	**SCF Energy diff. (kcal / mol)**
PBE-D3 H & F Opt	-4679.377405	-4679.474226	-60.76
PBE-D3 Full Cell Opt	-4679.988689	-4680.004556	-9.96
PBE0-TC-LRC-D3, R_C = 4 Å	-4677.885444	-4678.09188	-129.54
PBE0-TC-LRC-D3, R_C = 5 Å	-4677.706928	-4677.912856	-129.22
PBE0-TC-LRC-D3, R_C = 6 Å	-4677.602389	-4677.808255	-129.18
[Co(**L2**)–NBD][BAr$^F_4$]			
**Functional**	**Singlet SCF Energy (au)**	**Triplet SCF Energy (au)**	**SCF Energy diff. (kcal / mol)**
PBE-D3 H & F Opt	-4670.044946	-4669.964360	50.57
PBE-D3 Full Cell Opt	-4670.338849	-4670.258706	50.29
PBE0-TC-LRC-D3, R_C = 4 Å	-4668.422112	-4668.448147	-16.34
PBE0-TC-LRC-D3, R_C = 5 Å	-4668.245154	-4668.271166	-16.32
PBE0-TC-LRC-D3, R_C = 6 Å	-4668.140939	-4668.167248	-16.51

S.5.2. Molecular Calculations

Molecular calculations employed the GAUSSIAN 09 (Revision D.01) program package.[S24] Stuttgart-Dresden (SDD)[S25] relativistic effective core potentials (ECP) in combination with the associated basis sets were utilized to describe Co and P, with polarization functions added for P (ζ = 0.387).[S26] The 6-31G(d,p) basis sets[S27-28] were used on remaining atoms.

Electronic structure analyses were performed on the geometries of the Co cations extracted from the CP2K-optimised structures with the heavy atoms fixed at the experimental positions, allowing for disorder in the C_4 phosphine backbone of [Co(**L2**)–NBA][BAr$^F_4$] as described above. These structures had effective C_2 molecular symmetry. An electron density file suitable for further analysis was generated from a single-point calculation using the BP86 GGA functional[S29-30] in the triplet state.

The topology of the electron density was analysed by means of QTAIM (Quantum Theory of Atoms in Molecules),[S31] as implemented in the AIMALL package.[S32] Inner shell electrons on Co and P modelled by ECPs were represented by core density functions (extended wave-function format).

NBO calculations were performed using the NBO 6.0 program,[S33] using the same geometries as for the QTAIM calculations above. The Lewis structure was set using the $CHOOSE function to reflect the local C_2 symmetry of the [Co(**L2**)–NBA]$^+$ cation. In alpha spin this entailed 5 Co LPs and LPs on each P centre; in beta spin 3 Co LPs and 2 LPs on the P centres. In contrast, the Lewis structure determined by the program displayed 5 Co LPs, 1 P LP and one Co-P NBO in alpha spin, and 3 Co LPs and 2 Co-P NBOs in beta spin. The P LP NBOs are spatially related to Rh-P NBOs seen in previous analyses.[S19, 34]

NCI calculations were performed using the NCIPLOT program[S35-36], using the nearest neighbour ion-pair molecular structures extracted from CP2K optimised geometries. The promolecular electron density was employed.

NBO plots were created with Chemcraft[S37] with an outer contour value of 0.06. NLMO plots had an outer contour value of 0.03.

Optimising the two isolated cations using the PBE-D3 functional as either a singlet or a triplet yields structures different from the ones experimentally observed. The $[Co(\mathbf{L2})–NBA]^+$ cation adopts a square planar geometry very similar to its Rh analogue [S19] when optimised in the singlet state and adopts a pseudo-tetrahedral geometry when optimised as a triplet. A stationary point showing a 1,4-binding mode similar to that seen in the experimental crystal structure was located as a triplet but was shown to be a transition state linking two equivalent forms of the fully optimised triplet structure. The computed free energy barrier was 2 kcal/mol. The $[Co(\mathbf{L2})–NBD]^+$ cation adopts a distorted square planar geometry when optimised as a singlet and a pseudo-tetrahedral geometry when optimised as a triplet.

An NBA ligand binding free energy was estimated for the isolated triplet cation as 4 kcal/mol. This was derived by comparing free energies of the optimised structures of triplet $[Co(\mathbf{L2})]^+$ and free NBA with the triplet $[Co(\mathbf{L2})–NBA]^+$ cation.

S.5.3. Functional Testing and Spin Energetics.

Functional testing employed the GAUSSIAN 09 (revision D.01) program package[S24] using Stuttgart-Dresden (SDD)[S25] relativistic effective core potentials (ECP) in combination with the associated basis sets were utilized to describe Co and P, with polarization functions added for P (ζ = 0.387).[S26] The 6-31G(d,p) basis sets[S27-28] were used on the remaining atoms. The following different functionals were used: BP86,[S29-30] PBE,[S15-16] TPSS,[S38] B3LYP,[S39-40] PBE0,[S41-42] TPSSh,[S43-44] M06,[S45] M06L,[S46] B3PW91,[S39,S47-48] and ωB97X-D.[S49]

The analysis was based on the geometry of a Co cation extracted from the CP2K-optimised structures with the heavy atoms fixed at the experimental positions, except for any disorder on the phosphine backbone, as above, and computed in either the triplet or the singlet spin state. The resulting singlet-triplet energy difference can be seen in Figures S23 and S24. In the case of the $[Co(\mathbf{L2})–NBA]^+$ cation (Fig S23), all of the employed functionals clearly indicated it to be a triplet, with the singlet state even failing to converge with the three pure functionals. For the $[Co(\mathbf{L2})–NBD]^+$ system (Fig S24), the message is less clear. Pure functionals such as PBE predict it to be a singlet, whereas hybrid functionals tend to favour the triplet.

In order to investigate this further, we simplified both cations by truncating the cyclohexyl substituents on the phosphine ligands down to either methyls (R = Me) or hydrogens (R = H) and re-optimised each system whilst keeping the heavy-atom fixed geometry for the remaining atoms. The corresponding singlet-triplet energy gap was computed with B3LYP for the truncated $[Co(\mathbf{L2})–NBA]^+$ cations and BP86 for the truncated $[Co(\mathbf{L2})–NBD]^+$ cations, and found to be consistent with the un-truncated systems, as seen in Figures S23 and S24.

The truncated models were then used as input geometries for higher level DLPNO calculations, outlined below.

The DLPNO-CCSD(T)[S50-52] calculations were performed with the ORCA package (version 4.1.2)[S53-54] using the cc-pVTZ[S55-58] basis set on the truncated geometries. Initial single-point DFT calculations at the BP86/cc-pVTZ level were carried out in ORCA to yield Kohn-Sham determinants, which were then used as references for the DLPNO-CCSD(T). In these preliminary DFT calculations, the RI approximation was employed with the appropriate "def2/J" auxiliary basis-set,[S59] with the "Tight" SCF convergence criteria invoked and a Lebedev 434 integration grid used for the SCF iterations. For the DLPNO-CCSD(T) calculations, the "TightPNO" threshold setting was used, as has been demonstrated to be important to yield accuracy in computed spin-state energetics of cobalt complexes.[S60] Additionally, the corresponding auxiliary basis-set cc-pVTZ/C[S61-62] was employed for the correlation calculation for the DLPNO-CCSD(T) level.

The DLPNO calculations confirmed the earlier DFT results (Figs. S23 and S24) with the truncated [Co(**L2**)–NBA]$^+$ systems predicted to be triplets and the truncated [Co(**L2**)–NBD]$^+$ systems predicted to be singlets.

For the [Co(**L2**)–NBD]$^+$ cation, we therefore investigated the effects of small changes in geometry by rigidly rotating the alkene relative to the P-Co-P plane in small steps and computing the singlet-triplet gap for each step using the PBE0 functional. The result is plotted in Fig. S25. Inspecting the figure, starting from the experimental structure ($\phi = 28°$, where ϕ is the angle between the P-Co-P plane and the (C=C centroid)-Co-(C=C centroid) plane of the alkene) we find that a clockwise rotation of 15-20 degrees is sufficient to stabilise the singlet state versus the triplet.

S.6. Electronic Structure Analyses.

S.6.1. [Co(L2)–NBA][BAr^F$_4$]

Table S4. Key bond distances (Å) for fully optimised and heavy atoms fixed geometries, compared with experimental data. Crystallographic label provided in brackets if different to computational label.

Distance (Å)	Fully Optimised	Heavy Atom Fixed[a]	Experiment
Co – P1	2.238	2.232	2.2321(12)
Co – P2(P1')	2.238	2.232	2.2321(12)
Co – C1	3.178	2.612	2.612(15)
Co – C4(C1')	3.136	2.612	2.612(15)
Co – H11(H1A)	2.417	1.825	1.7758(8)
Co – H41(H1A')	2.370	1.824	1.7758(8)
C1 – H11(H1A)	1.105	1.123	0.990(13)
C1 – H12(H1B)	1.097	1.103	0.990(13)
C4(C1') – H41(H1A')	1.106	1.123	0.990(13)
C4(C1') – H42(H1B')	1.098	1.103	0.990(13)

[a]data are based on the experimental structure with only the positions of H and F atoms being optimised, with optimisation of the C_4 phosphine backbone under local C_2 symmetry.

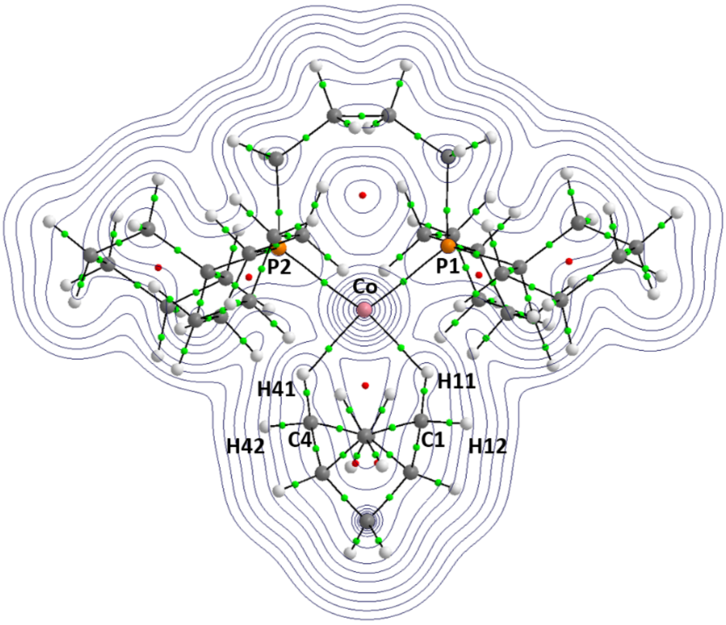

Figure S18: QTAIM molecular graph with density contours plotted in the Co-H11-H41 plane. Bond critical points (BCPs) are indicated in green and ring critical points (RCPs) are indicated in red.

Table S5. Key BCP metrics. Crystallographic label provided in brackets if different to computational label.

Bond	$\rho\ (r)$ /e.bohr^{-3}	$\nabla^2\ \rho\ (r)$ /e.bohr^{-5}	H (r)
Co – P1	0.085	0.212	-0.022
Co – P2(P1’)	0.085	0.212	-0.022
Co – H11(H1A)	0.047	0.190	-0.005
Co – H41(H1A’)	0.047	0.190	-0.005
C1 – H11(H1A)	0.252	-0.729	-0.235
C1 – H12(H1B)	0.271	-0.887	-0.267
C4(C1’) – H41(H1A’)	0.252	-0.729	-0.235
C4(C1’) – H42(H1B’)	0.271	-0.887	-0.267

Table S6. Key donor-acceptor interactions from the NBO second order perturbation analysis. Crystallographic label provided in brackets if different to computational label.

Donor	Acceptor	E(2) (kcal / mol)
α σ C1 – H11(H1A)	α Co LV	3.51
α Co LP	α σ* C1 – H11(H1A)	1.72
α P1 LP	α σ* C1 – H11(H1A)	1.18
α P2(P1’) LP	α σ* C1 – H11(H1A)	1.26
β σ C1 – H11(H1A)	β Co LV	5.88
β σ C1 – H11(H1A)	β Co LV	4.67
β P1 LP	β σ* C1 – H11(H1A)	0.98
β P2(P1’) LP	β σ* C1 – H11(H1A)	1.26
α σ C4(C1’) – H41(H1A’)	α Co LV	3.51
α Co LP	α σ* C4(C1’) – H41(H1A’)	1.72
α P1 LP	α σ* C4(C1’) – H11(H1A)	1.26
α P2(P1’) LP	α σ* C4(C1’) – H11(H1A)	1.18
β σ C4(C1’) – H41(H1A’)	β Co LV	5.88
β σ C4(C1’) – H41(H1A’)	β Co LV	4.67
β P1 LP	β σ* C4(C1’) – H41(H1A’)	1.26
β P2(P1’) LP	β σ* C4(C1’) – H41(H1A’)	0.98

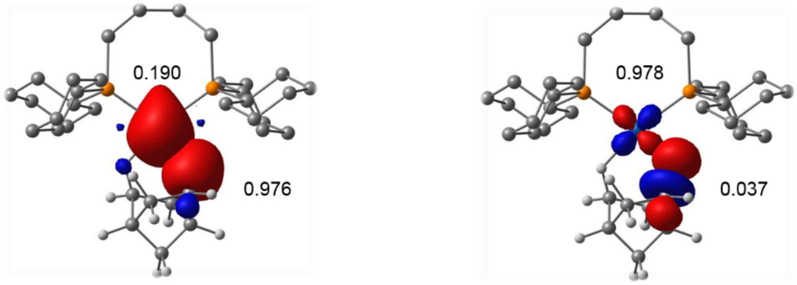

(a) α σ_{C1-H11} → α Co_{LV} (3.51 kcal/mol)

(b) α Co_{LP} → α σ^*_{C1-H11} (1.72 kcal/mol)

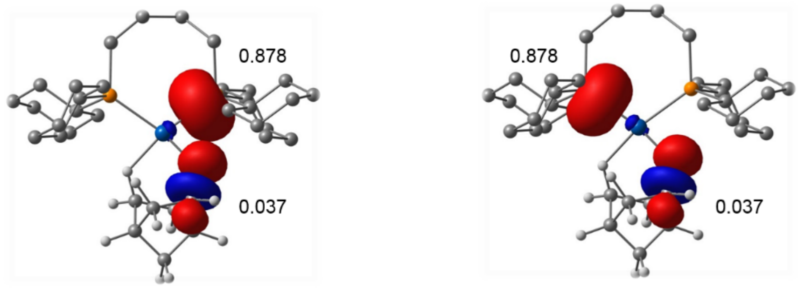

(c) α $P1_{LP}$ → α σ^*_{C1-H11} (1.18 kcal/mol)

(d) α $P2_{LP}$ → α σ^*_{C1-H11} (1.26 kcal/mol)

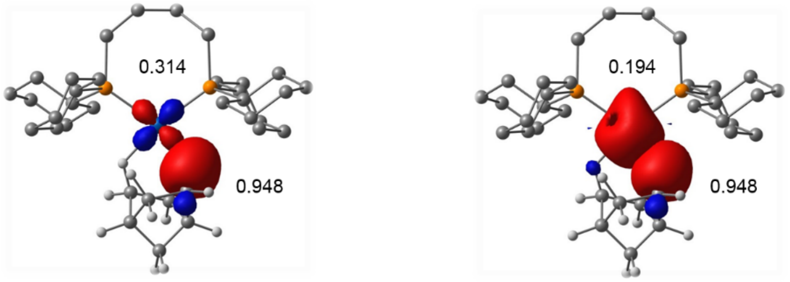

(e) β σ_{C1-H11} → β Co_{LV} (5.88 kcal/mol)

(f) β σ_{C1-H11} → β Co_{LV} (4.67 kcal/mol)

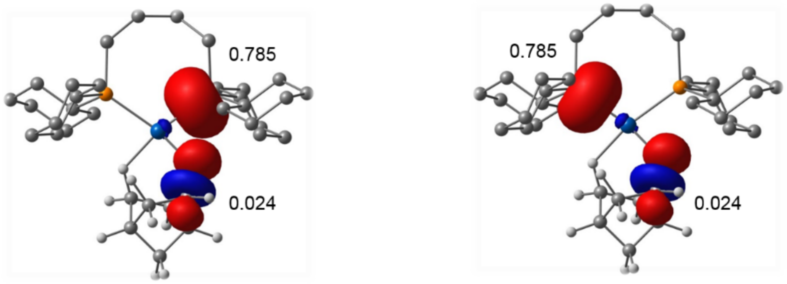

(g) β $P1_{LP}$ → β σ^*_{C1-H11} (0.98 kcal/mol)

(h) β $P2_{LP}$ → β σ^*_{C1-H11} (1.26 kcal/mol)

Figure S19. C1-H11 NBO donor-acceptor interactions with NBO occupancies as indicated.

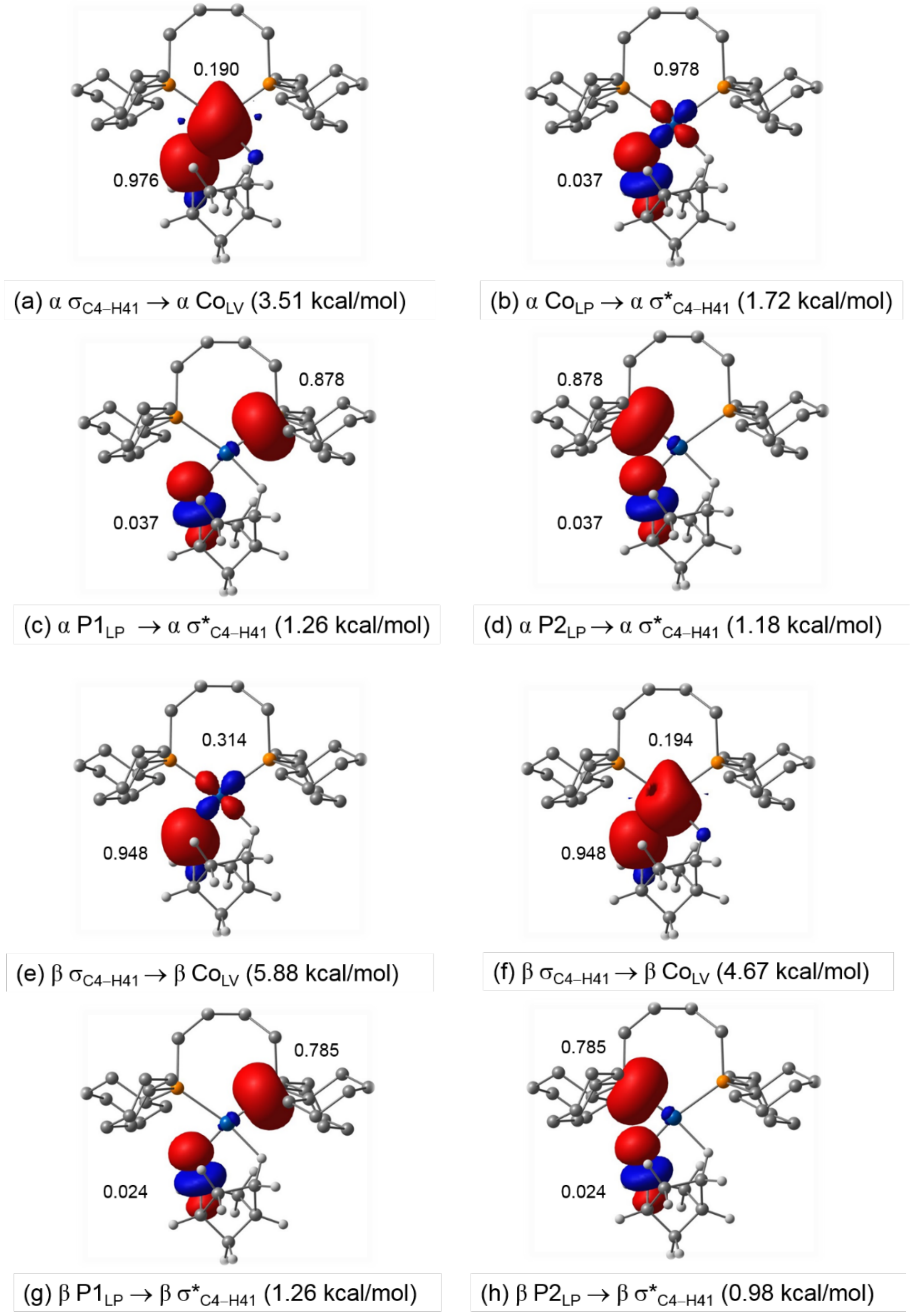

Figure S20. C4-H41 NBO donor-acceptor interactions with NBO occupancies as indicated.

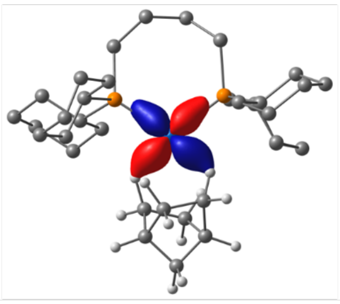

(a) α_{NLMO} 43 (97.8% Co)

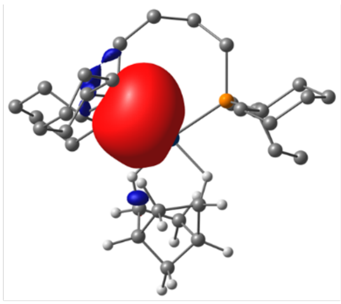

(b) α_{NLMO} 45 (87.2% P, 8.0 % Co, 1.4% C-H)

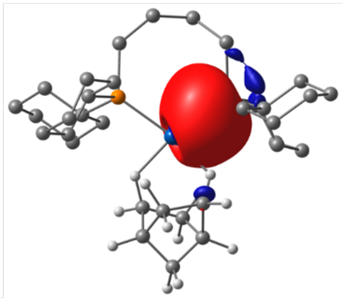

(c) α_{NLMO} 46 (87.2% P, 8.0 % Co, 1.4% C-H)

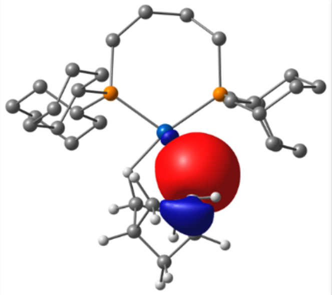

(d) α_{NLMO} 133 (97.0% C-H, 0.9 % Co)

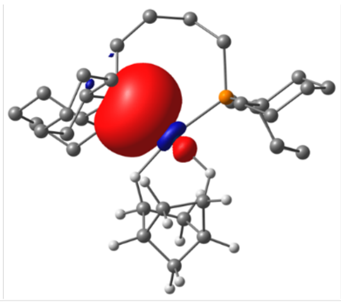

(a) β_{NLMO} 43 (76.8% P, 20.5 % Co)

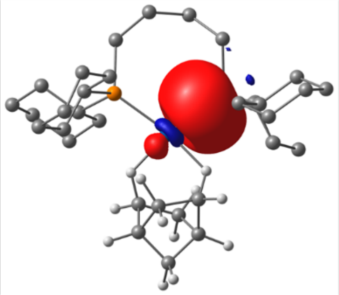

(b) β_{NLMO} 44 (76.8% P, 20.5 % Co)

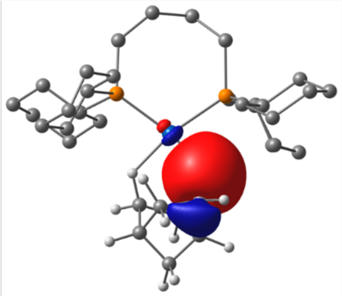

(c) β_{NLMO} 131 (94.6% C-H, 3.1 % Co)

Figure S21. Computed NLMOs involving the C1–H11 bond in both the α and β spins, with major contributions noted. A set of identical NLMOs is formed with the C4 – H41 bond.

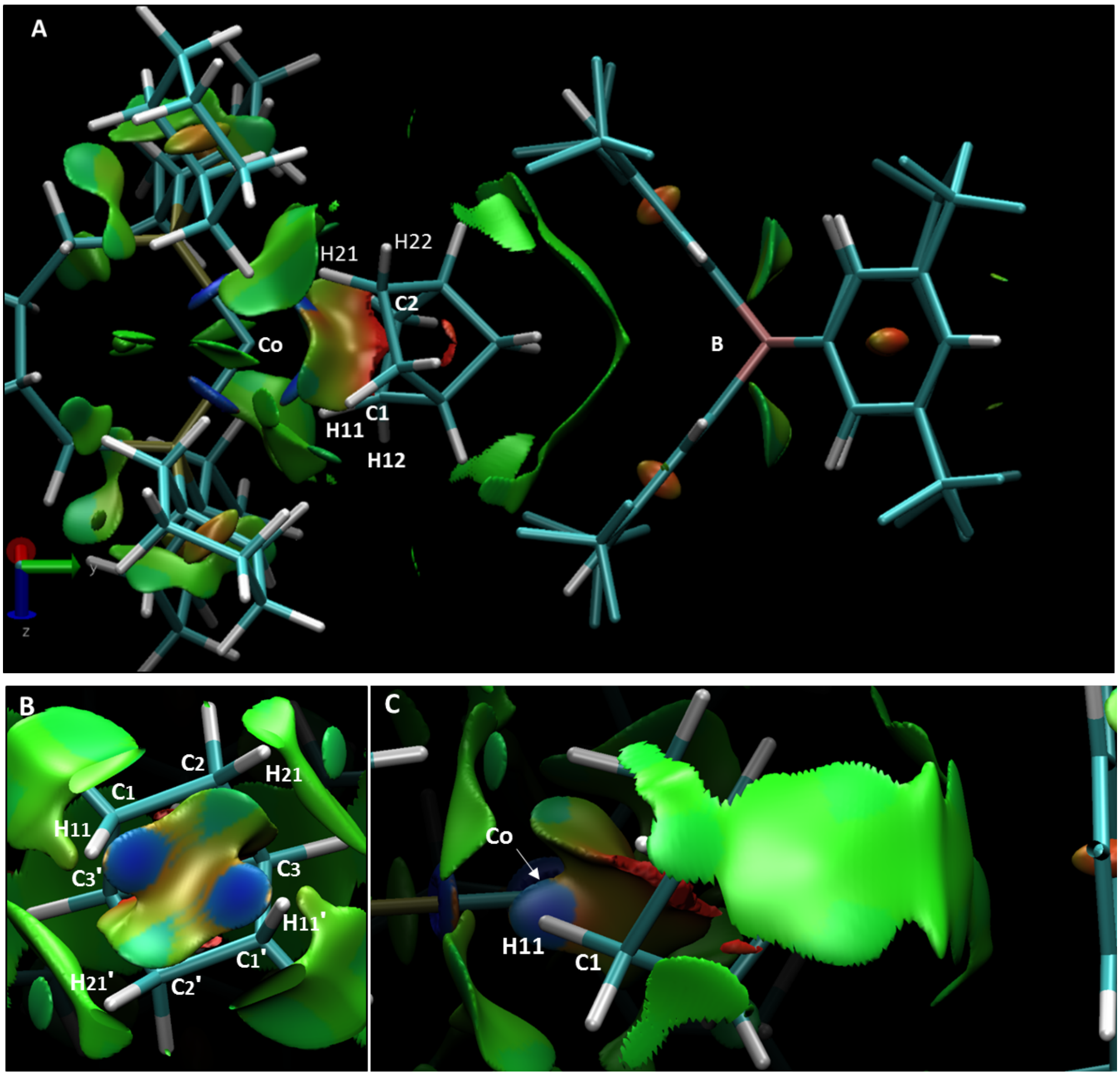

Figure S22. Views of the NCI plots of the heavy atom fixed [Co(**L2**)–NBA][BAr$^F_4$] ion-pair, (A) full ion pair from perpendicular to the Co···B axis; (B) NBA ligand from Co center; (C) down the H11···Co axis emphasizing the η^1-H···Co binding mode. Isosurfaces generated for s = 0.3 au and -0.07 < ρ < 0.07

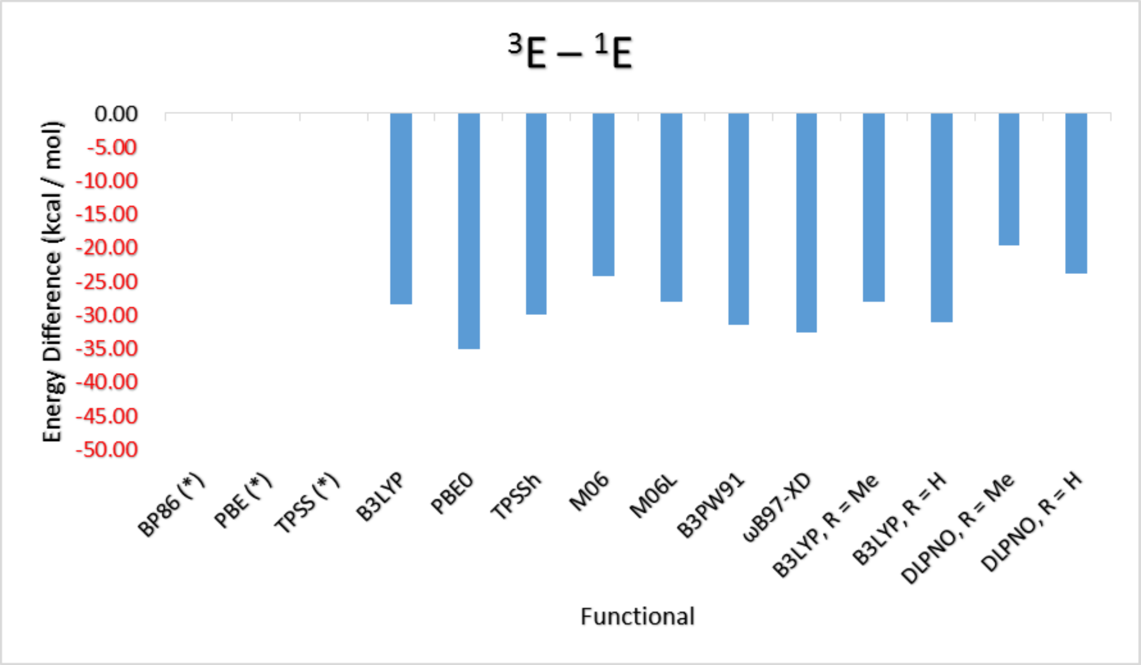

Figure S23. Triplet vs. singlet energy difference for the [Co(**L2**)–NBA]$^+$ cation using the indicated functionals. (*) The singlet spin state failed to converge.

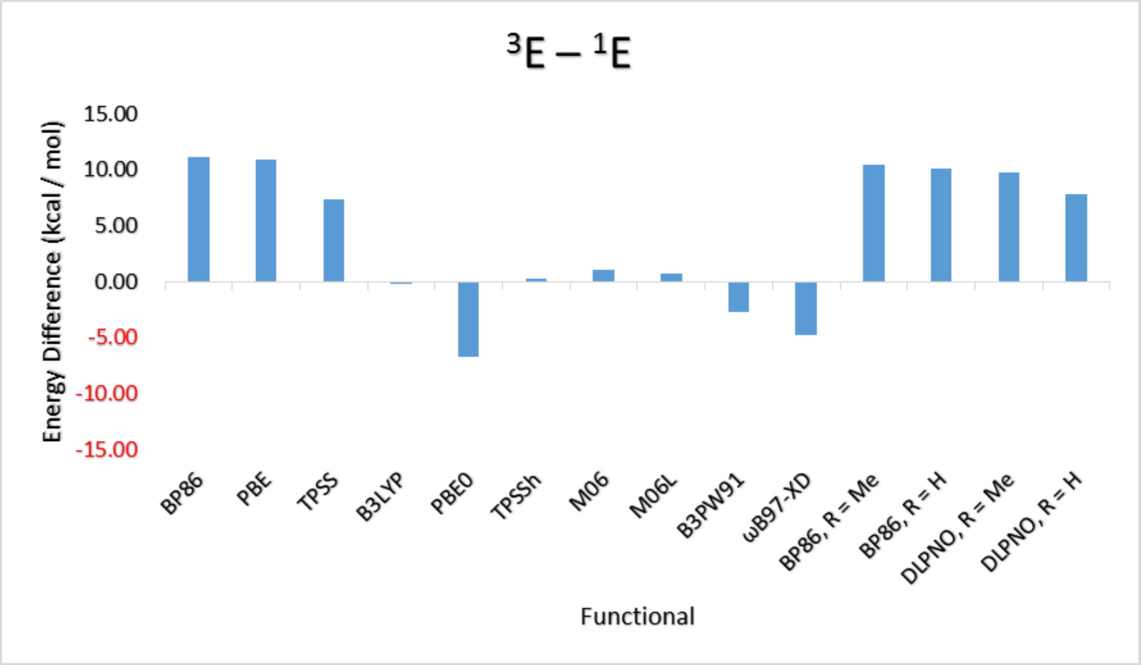

Figure S24. Triplet vs. singlet energy difference for the [Co(**L2**)–NBD]$^+$ cation using the indicated functionals.

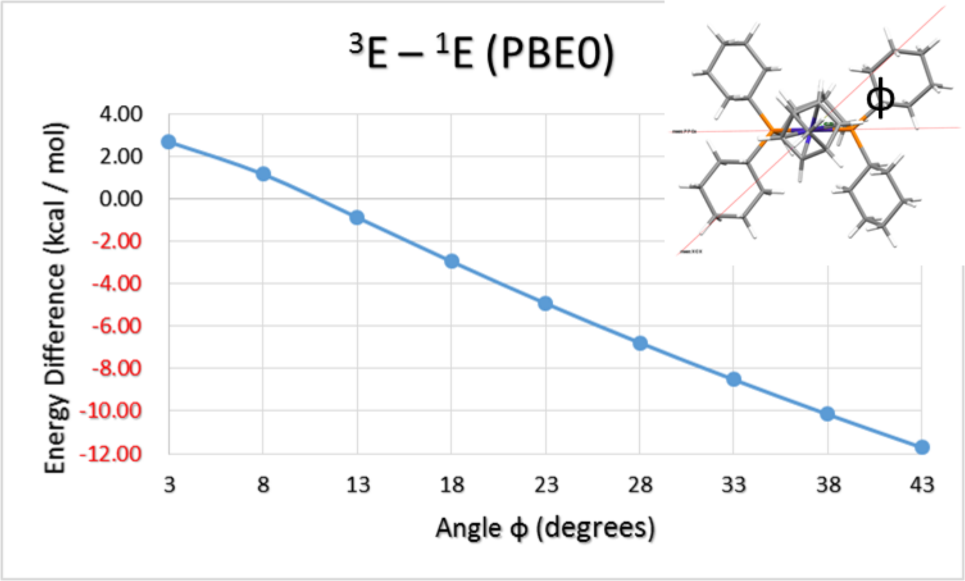

Figure S25. PBE0 Triplet vs. singlet energy difference for the [Co(**L2**)–NBD] cation as a function of a small rigid rotation of the alkene relative to the cobalt fragment. The angle phi (ϕ) is measured as the angle between the P-Co-P plane and the (C=C centroid)-Co-(C=C centroid) plane of the alkene, as seen in the inset. The experimental value is 28 degrees.

S.8. References

S1 A. J. Martínez-Martínez and A. S. Weller, *Dalt. Trans.*, 2019, **48**, 3551–3554.

S2 C. Piguet, *J. Chem. Educ.*, 1997, **74**, 815.

S3 F. M. Chadwick, A. I. McKay, A. J. Martinez-Martinez, N. H. Rees, T. Krämer, S. A. Macgregor and A. S. Weller, *Chem. Sci.*, 2017, **8**, 6014–6029.

S4 B. J. Cosier, A. M. A Glazer, *J. Appl. Crystallogr*. **1986**, 19, 105.

S5 Oxford Diffraction Ltd.; 2011.

S6 G. M. Sheldrick, *Acta Crystallogr. Sect. A Found. Crystallogr*. **2008**, 64, 112.

S7 G. M. Sheldrick, *Acta Crystallogr. Sect. A Found. Crystallogr*. **2015**, 71, 3.

S8 O. V. Dolomanov, L. J Bourhis, R. J Gildea, J. A. K. Howard, H. Puschmann, *J. Appl. Crystallogr.* **2009**, 42, 339.

S9 J. VandeVondele, M. Krack, F. Mohamed, M. Parrinello, T. Chassaing, J. Hutter, *Comput. Phys. Commun.* **2005**, *167*, 103-128.

S10 J. Hutter, M. Iannuzzi, F. Schiffmann, J. VandeVondele, *WIREs: Comput. Mol. Sci.* **2013**, *4*, 15-25.

S11 J. VandeVondele, J. Hutter, *J. Chem. Phys.* **2007**, *127*, 114105.

S12 C. Hartwigsen, S. Goedecker, J. Hutter, *Phys. Rev. B* **1998**, *58*, 3641-3662.

S13 S. Goedecker, M. Teter, J. Hutter, *Phys. Rev. B* **1996**, *54*, 1703-1710.

S14 M. Krack, *Theor. Chem. Acc.* **2005**, *114*, 145-152.

S15 J. P. Perdew, K. Burke and M. Ernzerhof, *Phys. Rev. Lett.* **1996**, 77, 3865-3868.

S16 J. P. Perdew, K. Burke and M. Ernzerhof, *Phys. Rev. Lett.* **1997**, 78, 1396-1396.

S17 Grimme, S.; Antony, J.; Ehrlich, S.; Krieg, H. *J. Chem. Phys.* **2010**, *132*, 154104.

S18 C. F. Macrae, I. J. Bruno, J. A. Chisholm, P. R. Edgington, P. McCabe, E. Pidcock, L. Rodriguez-Monge, R. Taylor, J. van de Streek, P. A. Wood, *J. Appl. Cryst.* **2008**, *41*, 466-470.

S19 A. J. Martínez-Martínez, B. E. Tegner, A. I. McKay, A. J. Bukvic, N. H. Rees, G. J. Tizzard, S. J. Coles, M. R. Warren, S. A. Macgregor, A. S. Weller, *J. Am. Chem. Soc.* **2018**, *140*, 14958-14970.

S20 J. Spencer and A. Alavi, *Phys. Rev. B* **2008**, 77, 193110.

S21 M. Guidon, F. Schiffmann, J. Hutter and J. VandeVondele, *J. Chem. Phys.* **2008**, 128, 214104.

S22 M. Guidon, J. Hutter and J. VandeVondele, *J. Chem. Theor. Comput.* **2009**, 5, 3010-3021.

S23 M. Guidon, J. Hutter and J. VandeVondele, *J. Chem. Theor. Comput.* **2010**, 6, 2348-2364.

S24 M. J. Frisch, G. W. Trucks, H. B. Schlegel, G. E. Scuseria, M. A. Robb, J. R. Cheeseman, G. Scalmani, V. Barone, B. Mennucci, G. A. Petersson, H. Nakatsuji, M. Caricato, X. Li, H. P. Hratchian, A. F. Izmaylov, J. Bloino, G. Zheng, J. L. Sonnenberg, M. Hada, M. Ehara, K. Toyota, R. Fukuda, J. Hasegawa, M. Ishida, T. Nakajima, Y. Honda, O. Kitao, H. Nakai, T. Vreven, J. A. Montgomery, J. E. Peralta, F. Ogliaro, M. Bearpark, J. J. Heyd, E. Brothers, K. N. Kudin, V. N. Staroverov, R. Kobayashi, J. Normand, K. Raghavachari, A. Rendell, J. C. Burant, S. S. Iyengar, J. Tomasi, M. Cossi, N. Rega, J. M. Millam, M. Klene, J. E. Knox, J. B. Cross, V. Bakken, C. Adamo, J. Jaramillo, R. Gomperts, R. E. Stratmann, O. Yazyev, A. J. Austin, R.

Cammi, C. Pomelli, J. W. Ochterski, R. L. Martin, K. Morokuma, V. G. Zakrzewski, G. A. Voth, P. Salvador, J. J. Dannenberg, S. Dapprich, A. D. Daniels, Farkas, J. B. Foresman, J. V. Ortiz, J. Cioslowski, D. J. Fox, Gaussian Inc., Wallingford CT, **2013**.

S25 D. Andrae, U. Häußermann, M. Dolg, H. Stoll, H. Preuß, *Theor. Chim. Acta* **1990**, *77*, 123-141.

S26 A. Höllwarth, M. Böhme, S. Dapprich, A. W. Ehlers, A. Gobbi, V. Jonas, K. F. Köhler, R. Stegmann, A. Veldkamp, G. Frenking, *Chem. Phys. Lett.* **1993**, *208*, 237-240.

S27 W. J. Hehre, R. Ditchfield, J. A. Pople, *J. Chem. Phys.* **1972**, *56*, 2257-2261.

S28 P. C. Hariharan, J. A. Pople, *Theor. Chim. Acta* **1973**, *28*, 213-222.

S29 A. D. Becke, *Phys. Rev. A* **1988**, 38, 3098-3100.

S30 J. P. Perdew, *Phys. Rev. B* **1986**, 33, 8822-8824.

S31 R. F. W. Bader, *Atoms in Molecules: A Quantum Theory*, Clarendon Press, **1994**.

S32 T. A. Keith, TK Gristmill Software, Overland Park KS, USA, **2017**.

S33 E. D. Glendening, J. K. Badenhoop, A. E. Reed, J. E. Carpenter, J. A. Bohmann, C. M. Morales, C. R. Landis, F. Weinhold, Theoretical Chemistry Institute, University of Wisconsin, Madison, WI, **2013**.

S34 S. D. Pike, F. M. Chadwick, N. H. Rees, M. P. Scott, A. S. Weller, T. Krämer, S. A. Macgregor, *J. Am. Chem. Soc.* **2015**, *137*, 820-833.

S35 E. R. Johnson, S. Keinan, P. Mori-Sánchez, J. Contreras-García, A. J. Cohen, W. Yang, *J. Am. Chem. Soc.* **2010**, *132*, 6498-6506.

S36 J. Contreras-Garcia, E. R. Johnson, S. Keinan, R. Chaudret, J-P. Piquemal, D. N. Beratan, and W. Yang. *J. Chem. Theory Comput.* **2011**, 7, 625-632.

S37 Chemcraft - graphical software for visualization of quantum chemistry computations. https://www.chemcraftprog.com

S29 A. D. Becke, *Phys. Rev. A*, **1988**, 38, 3098-3100.

S30 J. P. Perdew, *Phys. Rev. B*, **1986**, 33, 8822-8824.

S15 J. P. Perdew, K. Burke and M. Ernzerhof, *Phys. Rev. Lett.*, **1996**, 77, 3865-3868.

S16 J. P. Perdew, K. Burke and M. Ernzerhof, *Phys. Rev. Lett.*, **1997**, 78, 1396-1396.

S38 J. Tao, J. P. Perdew, V. N. Staroverov and G. E. Scuseria, *Phys. Rev. Lett.*, **2003**, 91, 146401.

S39 A. D. Becke, *J. Chem. Phys*, **1993**, 98, 5648-5652.

S40 C. Lee, W. Yang and R. G. Parr, *Phys. Rev. B*, **1988**, 37, 785-789.

S41 C. Adamo and V. Barone, *J. Chem. Phys.*, **1999**, 110, 6158-6170.

S42 M. Ernzerhof and G. E. Scuseria, *J. Chem. Phys.*, **1999**, 110, 5029-5036.

S43 V. N. Staroverov, G. E. Scuseria, J. Tao and J. P. Perdew, *J. Chem. Phys.*, **2003**, 119, 12129-12137.

S44 V. N. Staroverov, G. E. Scuseria, J. Tao and J. P. Perdew, *J. Chem. Phys.*, **2004**, 121, 11507-11507.

S45 Y. Zhao and D. G. Truhlar, *Theor. Chem. Account.*, **2008**, 120, 215-241.

S46 Y. Zhao and D. G. Truhlar, *J. Chem. Phys.*, **2006**, 125, 194101.

S47 J. P. Perdew, J. A. Chevary, S. H. Vosko, K. A. Jackson, M. R. Pederson, D. J. Singh and C. Fiolhais, *Phys. Rev. B*, **1992**, 46, 6671-6687.

S48 J. P. Perdew, J. A. Chevary, S. H. Vosko, K. A. Jackson, M. R. Pederson, D. J. Singh and C. Fiolhais, *Phys. Rev. B*, **1993**, 48, 4978-4978.

S49 J.-D. Chai and M. Head-Gordon, *Phys. Chem. Chem. Phys.*, **2008**, 10, 6615-6620.
S50 C. Riplinger and F. Neese, *J. Chem. Phys.* **2013**, 138, 034106.
S51 C. Riplinger, B. Sandhoefer, A. Hansen and F. Neese, *J. Chem. Phys.* **2013**, 139, 134101.
S52 M. Saitow, U. Becker, C. Riplinger, E. F. Valeev, F. Neese, *J. Chem. Phys.* **2017**, *146*, 164105.
S53 F. Neese, *WIREs: Comput. Mol. Sci*, **2012**, 2, 73-78.
S54 F. Neese, *WIREs: Comput. Mol. Sci*, **2018**, 8, e1327.
S55 N. B. Balabanov and K. A. Peterson, *J. Chem. Phys.*, **2005**, 123, 064107.
S56 N. B. Balabanov and K. A. Peterson, *J. Chem. Phys.*, **2006**, 125, 074110.
S57 T. H. Dunning, *J. Chem. Phys.*, **1989**, 90, 1007-1023.
S58 D. E. Woon and T. H. Dunning, *J. Chem. Phys.*, **1993**, 98, 1358-1371.
S59 F. Weigend, *Phys. Chem. Chem. Phys.* **2006**, *8*, 1057-1065.
S60 S. E. Neale, Private communication.
S61 J. G. Hill, J. A. Platts, *J. Chem. Phys.* **2008**, *128*, 044104.
S62 F. Weigend, A. Köhn, C. Hättig, *J. Chem. Phys.* **2002**, *116*, 3175-3183.
